# Role of cytoplasmic localization of maspin in promoting cell invasion in breast cancer with aggressive phenotype

**DOI:** 10.1038/s41598-021-90887-z

**Published:** 2021-05-31

**Authors:** Tomohiko Sakabe, Makoto Wakahara, Goshi Shiota, Yoshihisa Umekita

**Affiliations:** 1 Department of Pathology, Faculty of Medicine, Tottori University, Yonago, 683-8503 Japan; 2 Division of General Thoracic Surgery and Breast and Endocrine Surgery, Department of Surgery, Faculty of Medicine, Tottori University, Yonago, 683-8503 Japan; 3 Division of Medical Genetics and Regenerative Medicine, Department of Genomic Medicine and Regenerative Therapy, Faculty of Medicine, Tottori University, Yonago, 683-8503 Japan

**Keywords:** Epithelial-mesenchymal transition, Breast cancer

## Abstract

Mammary serine protease inhibitor (maspin) is a tumor suppressor gene that is downregulated during carcinogenesis and breast cancer progression. While the nuclear localization of maspin is essential for tumor suppression, we previously reported that the cytoplasmic localization of maspin was significantly correlated with poor prognosis in breast cancer patients. To understand the mechanisms that underlie oncogenic role of cytoplasmic maspin, we studied its biological function in breast cancer cell lines. Subcellular localization of maspin in MDA-MB-231 breast cancer cells was mainly detected in the cytoplasm, whereas in MCF10A mammary epithelial cells, maspin was present in both cytoplasm and nucleus. In MDA-MB-231 cells, maspin overexpression promoted cell proliferation and cell invasion, whereas maspin downregulation resulted in the opposite effect. Further, we observed that SRGN protein levels were increased in MDA-MB-231 cells stably overexpressing maspin. Finally, maspin overexpression in MDA-MB-231 cells resulted in the N-cadherin and epithelial mesenchymal transition (EMT)-related transcription factors upregulation, and TGFβ signaling pathway activation. These results suggested that cytoplasmic maspin enhances the invasive and metastatic potential in breast cancer cells with aggressive phenotype by inducing EMT via SRGN/TGFβ axis. This study demonstrated a novel biological function of cytoplasmic maspin in progression of breast cancer cells with an aggressive phenotype.

## Introduction

Breast cancer is the most common incident cancer in women and the leading cause of cancer death worldwide^1^. Additionally, breast cancer usually exhibits high heterogeneity at the molecular level and a differential therapeutic response in accordance with their distinct molecular profiles. According to the current classification for breast cancer subtypes proposed at St. Gallen Conferences, breast cancer is classified into the following four subtypes: (1) triple-negative, which is defined as estrogen receptor (ER), progesterone receptor (PR), and human epidermal growth factor receptor 2 (HER2)-negative; (2) HER2-enriched, which is defined as HER2-positive; (3) luminal A-like, which is defined as ER-positive and/or PR-positive, HER2-negative, low Ki67 labeling index; and (4) luminal B-like, which is defined as ER-positive and/or PR-positive, HER2-negative, and high Ki67 labeling index^2^. In particular, triple-negative breast cancer (TNBC) subtype exhibits an aggressive phenotype that is characterized by rapid tumor growth, easy metastasis, early recurrence, lack of effective targeted therapies, and poor prognosis^3,4^. Therefore, increasing attention has been focused on the mechanisms that allow breast cancer cells to acquire their malignant and aggressive phenotype.

Human mammary serine protease inhibitor (maspin), which is located at chromosome 18q21.3 and encoded by the SERPINB5 gene, was originally identified in human normal mammary epithelial cells^5–8^. Although maspin is structurally an unusual member of the serine protease inhibitor (serpin) superfamily, no proteases have been found to be inhibited by maspin through a canonical serpin mechanism. Since its expression was reportedly reduced or absent during carcinogenesis and progression of ductal carcinoma, maspin has been considered a tumor suppressor that induced tumor cell apoptosis^9^, suppression of cellular motility^10,11^, and regulation of drug sensitivity^12,13^. Thus, there are many studies focusing on the clinicopathological features and prognostic influence of maspin expression in various types of cancer, such as breast, lung, prostate, and pancreatic cancer; however, it remains controversial whether maspin expression is an unfavorable or favorable indicator in patients with several types of cancer^14^. We reported that cytoplasmic expression of maspin is an important indicator of unfavorable prognosis in patients with breast and lung cancer^15–17^. Similarly, other groups also described that cytoplasmic localization of maspin is closely associated with poor prognosis in patients with non-small cell lung cancer and laryngeal carcinoma^18,19^. In addition, Goulet et al*.* reported that nuclear localization of maspin is indispensable to its tumor suppressive function^20^. Based on these findings, we hypothesized that maspin has different functions in carcinogenesis and tumor progression, which depend on its subcellular localization, and that cytoplasmic maspin might positively contribute to the acquisition of malignant and aggressive phenotype in cancer cells. To our knowledge, no studies have examined the oncogenic role of maspin in breast cancer cells, therefore; we investigated the biological function of cytoplasmic maspin in breast cancer cells.

## Results

### Subcellular localization of maspin in normal and breast cancer cell lines

**Figure 1:**
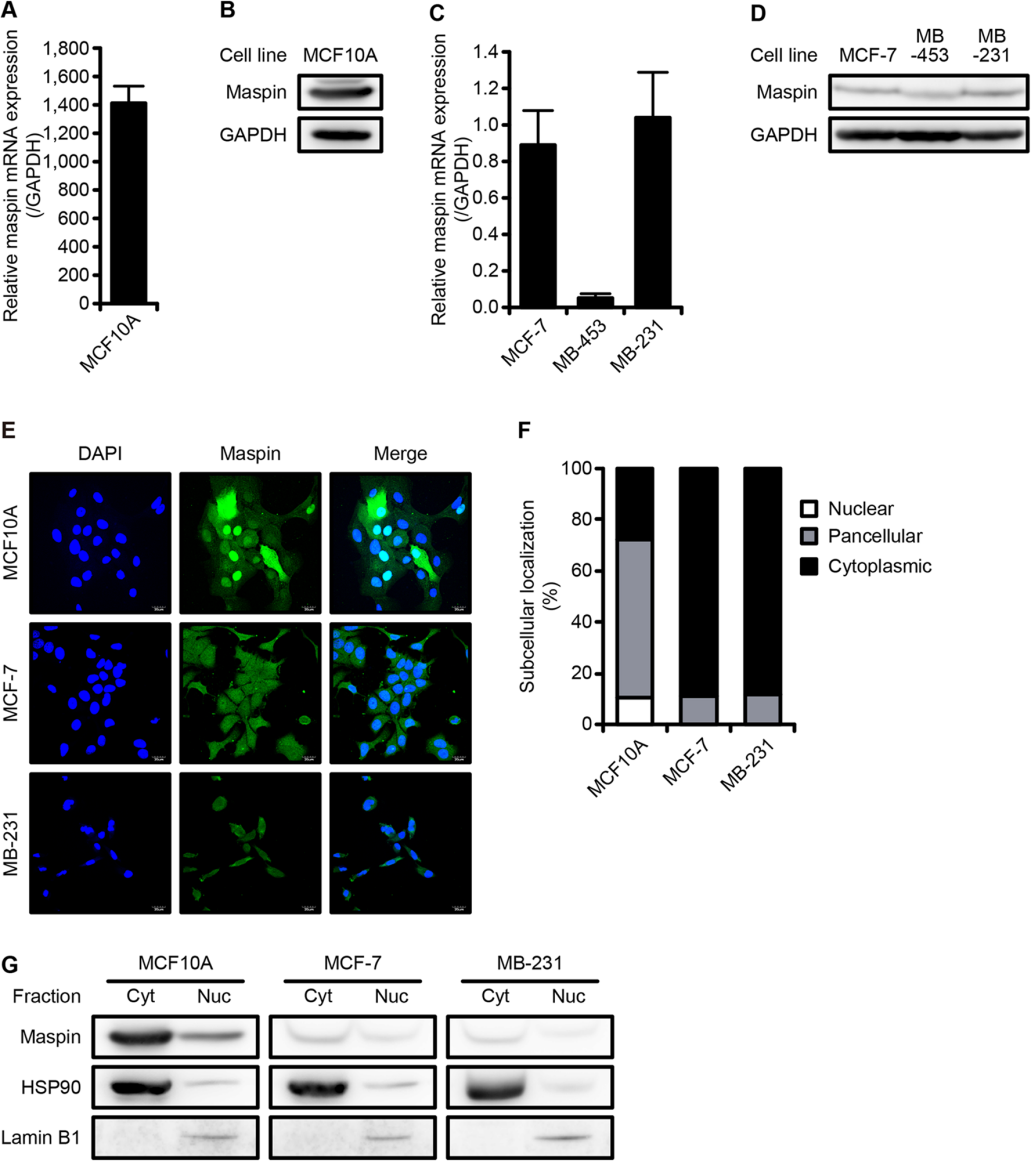
Expression and subcellular localization of maspin in non-tumorigenic and tumorigenic cell lines. (**A**) Relative mRNA expression level of maspin in MCF10A cells. mRNA expression was normalized to level of *GAPDH*. Data are shown as the mean ± SD (*n* = 3). (**B**) Protein expression level of maspin in MCF10A cells. GAPDH is shown for loading control. Whole western blots are presented in Supplementary Fig. S7. (**C**) Relative mRNA expression levels of maspin in breast cancer cell lines. mRNA expression was normalized to levels of *GAPDH*. Data are shown as the mean ± SD (*n* = 3). (**D**) Protein expression levels of maspin in three breast cancer cell lines. Expression level of GAPDH was used as a protein loading control. Whole western blots are presented in Supplementary Fig. S8. (**E**) Representative images of subcellular localization of maspin protein in MCF10A, MCF-7, and MDA-MB-231 cells. Expression of maspin protein was detected using anti-maspin antibody (clone G167-70). Nuclei was counterstained with DAPI. Scale bars represent 20 μm. Images were acquired using Olympus FLUOVIEW FV1000D confocal laser scanning microscope and Olympus FV10-ASW software (ver. 02.01). (**F**) Quantification of the subcellular localization of maspin. Bar graph shows the percentage of maspin distribution that is calculated by counting number of cells exhibiting pancellular, nuclear, or cytoplasmic distribution (*n* = 3). (**G**) Protein expression of maspin in the cytoplasmic (Cyt) and nuclear (Nuc) fractions. Expression levels of HSP90 and Lamin B1 was used as protein loading control in the cytoplasmic and nuclear fractions, respectively. Whole western blots are presented in Supplementary Fig. S9.

In our previous papers, we reported that cytoplasmic localization of maspin was related to the poor prognosis in breast cancer patients^21^. To elucidate the molecular mechanism underlying its role in breast cancer progression, we first investigated maspin expression in a non-tumorigenic human mammary epithelial cell line (MCF10A), a luminal breast cancer cell line (MCF-7), and two TNBC cell lines (MDA-MB-453, MDA-MB-231). In MCF10A cells, maspin was abundantly expressed at both the mRNA and protein levels (Fig. 1A,B). In three breast cancer cell lines, maspin mRNA and protein expression were narrowly detected (Fig. 1C,D). Since maspin in MDA-MB-453 cells exhibited a slightly lower expression level as compared with other cell lines, MCF10A, MCF-7, and MBA-MB-231 cell lines were used for the subsequent analysis. Next, immunofluorescence with the different antibodies of the two clones was performed in order to determine the subcellular localization of maspin. Maspin protein was localized to both nucleus and cytoplasm (pan-cellular) in MCF10A cells, whereas maspin protein signal was mostly detected in the cytoplasm of MCF-7 and MDA-MB-231 cells (Fig. 1E,F, Supplementary Fig. S1). Additionally, similar results to the immunofluorescence were also obtained in the nuclear/cytoplasmic fractionation assay (Fig. 1G). These results indicate that in breast cancer cell lines, cytoplasm-to-nucleus transport of this protein is disrupted.

### Role of maspin expression and subcellular localization in proliferation of normal and breast cancer cell lines

**Figure 2:**
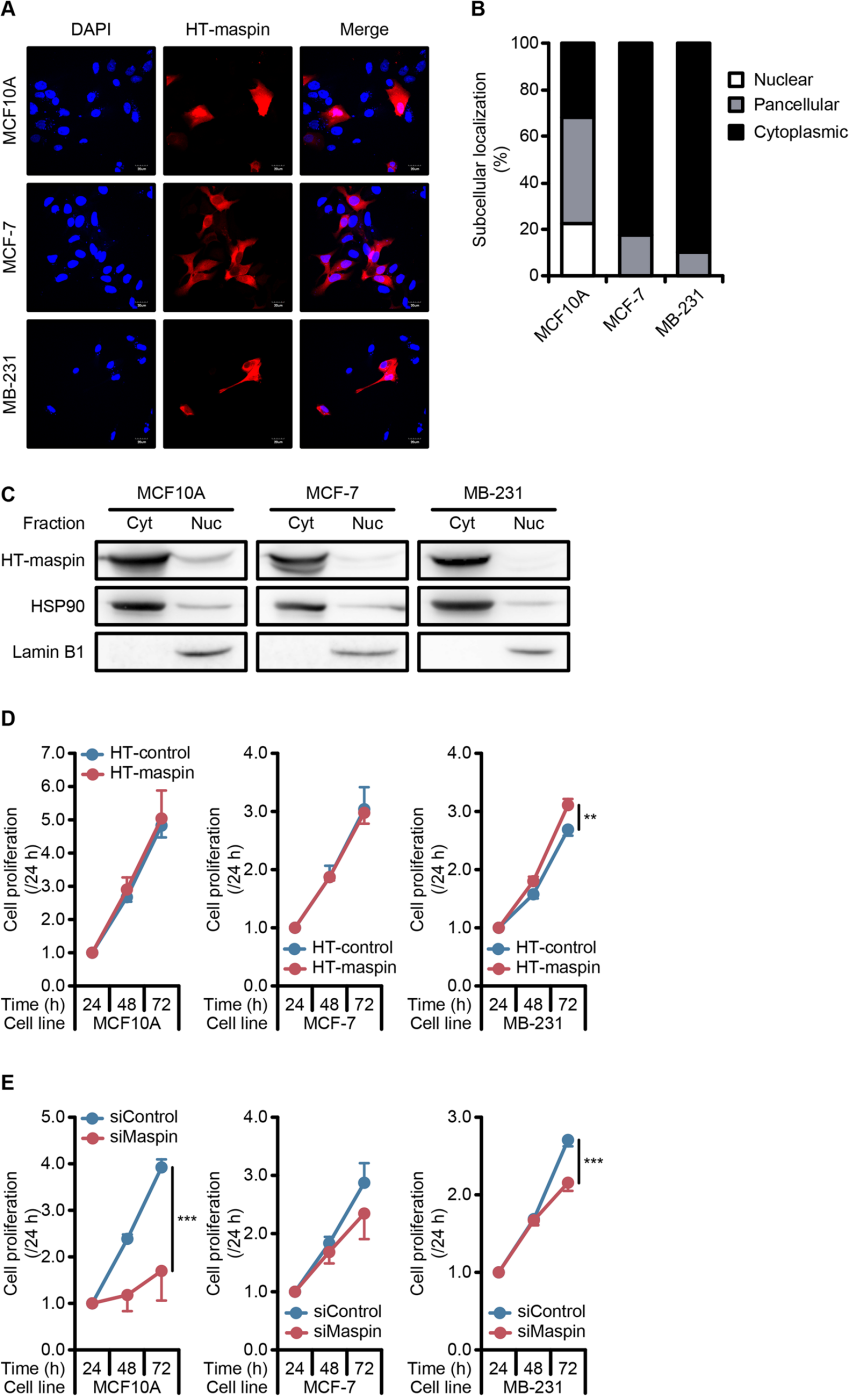
The relevance of maspin expression and subcellular localization to cell proliferation. (**A**) Representative images of subcellular localization of HaloTag-fused maspin. Nuclei was counterstained with DAPI. Scale bars represent 20 μm. Images were acquired using Olympus FLUOVIEW FV1000D confocal laser scanning microscope and Olympus FV10-ASW software (ver. 02.01). (**B**) Quantification of the subcellular localization of HaloTag-fused maspin. Bar graph shows the percentage of HaloTag-fused maspin distribution that is calculated by counting number of cells exhibiting pancellular, nuclear, or cytoplasmic distribution (*n* = 5, 6). (**C**) Protein expression of HaloTag-fused maspin in the cytoplasmic (Cyt) and nuclear (Nuc) fractions. Expression levels of HSP90 and Lamin B1 was used as protein loading control in the cytoplasmic and nuclear fractions, respectively. Whole western blots are presented in Supplementary Fig. S10. (**D**) Plots show cell proliferation of cells overexpressing HaloTag-fused maspin or HaloTag-control. The data are normalized to the corresponding value of 24 h. Data are shown as the mean ± SD (*n* = 4). **P < 0.01; Student’s *t*-test. (**E**) Plot shows cell proliferation of cells expressing maspin siRNA or control siRNA. The data are normalized to the corresponding value of 24 h. Data are shown as the mean ± SD (*n* = 4). ***P < 0.001; Student’s *t*-test.

Since maspin reportedly plays a pivotal role in the suppression of several cancers^9–13^, we aimed to clarify whether expression and localization of maspin may affect cell proliferation in normal and breast cancer cell lines. MCF10A, MCF-7, and MDA-MB-231 cells transiently transfected with plasmid DNA encoding HaloTag-fused maspin showed a remarkable increase in maspin mRNA levels (Supplementary Fig. S2A–C), and HaloTag-fused maspin proteins were detected in these cells as well (Supplementary Fig. S2D). As with the subcellular localization of the endogenous maspin, HaloTag-fused maspin showed a pan-cellular localization in MCF10A cells, whereas in MCF-7 and MDA-MB-231 cells this protein was expressed mainly in the cytoplasm (Fig. 2A,B). In addition, these subcellular localizations were also confirmed in the fractionation assay (Fig. 2C). Although cell proliferation capability was not changed in MCF10A and MCF-7 cells, it was slightly increased in MDA-MB-231 cells expressing HaloTag-fused maspin as compared with that in the control cells (Fig. 2D). Next, we performed siRNA-mediated maspin knock down in MCF10A, MCF-7, and MDA-MB-231 cells. Specific siRNA targeting maspin effectively knocked down both maspin mRNA (Supplementary Fig. S3A–C) and protein expression (Supplementary Fig. S3D). siRNA-mediated suppression of maspin expression resulted in significantly decreased cell proliferation in MCF10A cells (Fig. 2E). In breast cancer cells, cell proliferation of MDA-MB-231 cells was also significantly decreased by maspin knockdown, whereas cell growth of MCF-7 cells slightly decreased, indicating that cytoplasmic localization of maspin is correlated with cell proliferation capability regardless of tumorigenicity.

### Role of cytoplasmic localization of maspin in the promotion of MDA-MB-231 cell invasion

**Figure 3:**
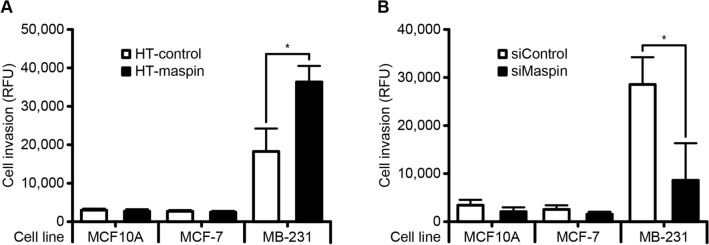
The relevance of maspin expression and subcellular localization to cell invasion. (**A**) Cell invasion capability of cells overexpressing HaloTag-fused maspin or HaloTag-control. Data are shown as the mean ± SD (*n* = 3). *P < 0.05; Student’s *t*-test. (**B**) Cell invasion capability of cells expressing maspin siRNA or control siRNA. Data are shown as the mean ± SD (*n* = 3). *P < 0.05; Student’s *t*-test.

To gain further insight into the precise role of cytoplasmic maspin in tumor progression, we examined the correlation between cytoplasmic maspin expression and cell invasion capability. The cell invasion was significantly increased in response to overexpression of HaloTag-fused maspin protein in MDA-MB-231 cells, but not in MCF10A and MCF-7 cells (Fig. 3A). Furthermore, the opposite result was also observed by suppression of maspin in these cell lines (Fig. 3B). It has been reported that nuclear translocation of maspin protein is required for the its tumor suppressive activity^20,22^. Therefore, to further investigation the effect of subcellular localization of maspin on the invasiveness of MDA-MB-231 cells, we constructed HaloTag and nuclear localization signal (NLS)-fused maspin expressed in both the nucleus and cytoplasm of MCF-7 and MDA-MB-231 cells (Supplementary Fig. S4A–C). As reportedly, expression of maspin in both the nucleus and cytoplasm significantly reduced the invasive capability of MDA-MB-231 cells (Supplementary Fig. S4D). These findings indicate that cytoplasmic maspin expression is closely correlated with the promotion of invasive behavior in some breast cancer cells, such as MDA-MB-231 cells.

### Identification and analysis of gene signature associated with maspin overexpression in MDA-MB-231 cells

**Figure 4:**
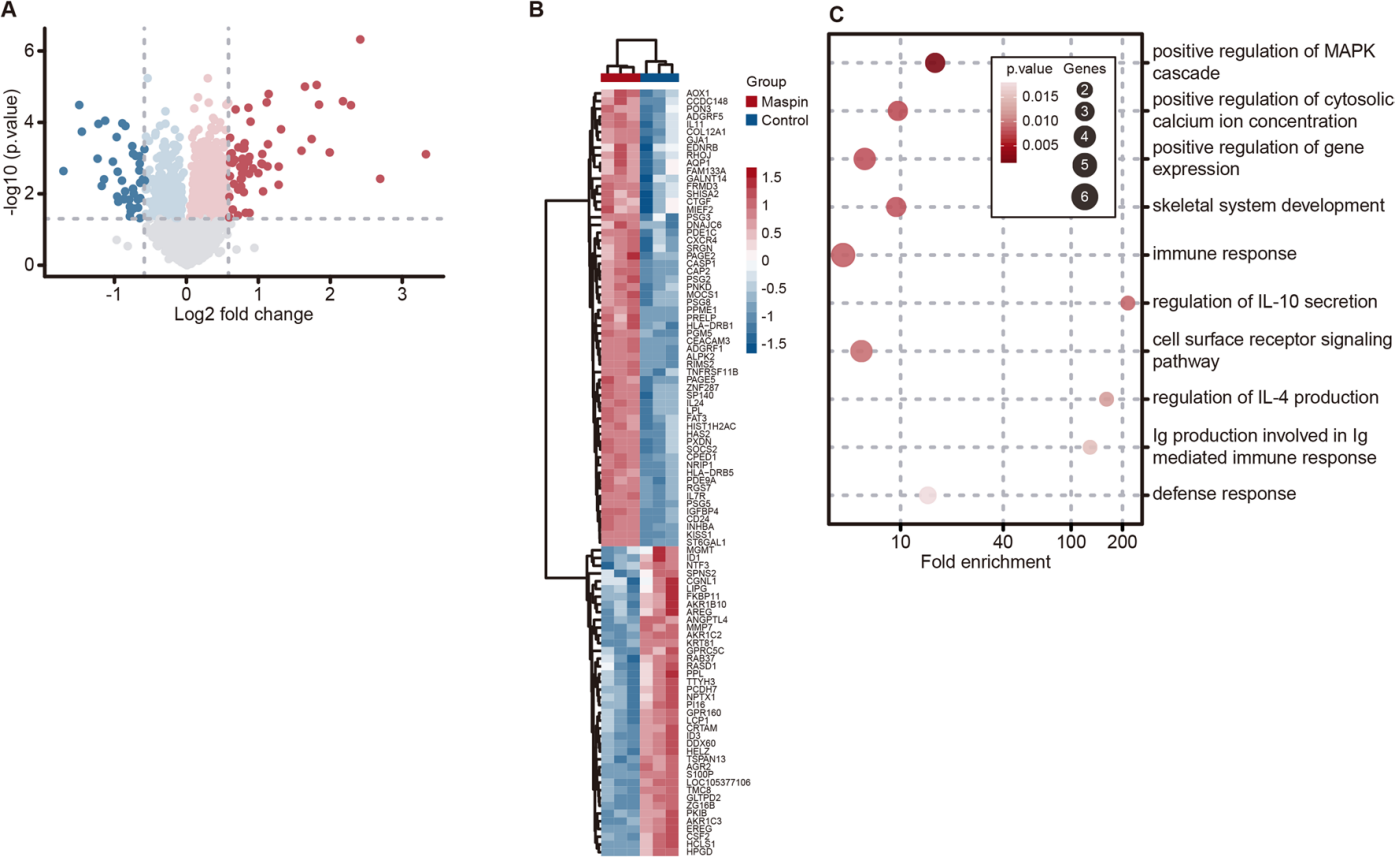
Analysis of the gene expression profile in MDA-MB-231 cells stably expressing maspin or control. (**A**) Comparison of mRNA expression profiles between MB-231-maspin and MB-231-control cells. Volcano plot shows expression levels upregulated (red dot), downregulated (blue dot), and unchanged (gray dot) in MB-231-maspin cells. The x-axis is log2 fold change of gene expression levels between to cell lines, and the y-axis is adjusted *P*-value based on -log10. Red and blue dots show the differentially expressed genes (DEGs) based on *P* < 0.05. Deep color represents 1.5-fold expression difference. (**B**) Heatmap and hierarchical clustering of the DEGs with > 1.5-fold change in MB-231-maspin cells. (**C**) Gene ontology (GO) enrichment analysis based on the DAVID biological process term. Bubble chart represents top-10 cluster enriched in a list of iDEGs. The x-axis is fold enrichment values, and the y-axis is the enrichment term. The size of bubble represents the number of DEGs. Transparency of the bubbles represents *P*-value. R (ver. 4.0.2, https://www.r-project.org)^23^ and RStudio (Ver. 1.3.959, https://rstudio.com) software were used to plot volcano plot, heatmap, and bubble chart. Tidyverse packages (ver. 1.3.0, https://www.tidyverse.org)^24^ and pheatmap (ver. 1.0.12, https://CRAN.R-project.org/package=pheatmap)^25^ packages were used to run the software.

A stable cell line, MB-231-maspin, was established. This line stably overexpressed maspin mRNA and protein as compared with the control cell line, MB-231-control as detected by reverse-transcription quantitative PCR (RT-qPCR) and western blot (Supplementary Fig. S5). To examine the putative mechanism underlying cytoplasmic maspin-dependent promotion of cell invasion, we performed a comparison of gene expression profiles in these two established cell lines by DNA microarray. Consequently, a total of 1866 genes were observed as significantly differentially expressed genes (DEGs) in MB-231-maspin versus MB-231-control cells (Fig. 4A, Supplementary Table S1). Eventually, a total of 99 genes, of which 59 genes were induced (induced differentially expressed genes: iDEGs) and 40 genes were repressed (repressed differentially expressed genes: rDEG), were designated based on a threshold of *P*-values < 0.05 and |logFC| > 0.585, corresponding to a fold change > 1.5 or < 1/1.5, as candidate DEGs (Fig. 4B). Gene ontology (GO) terms enrichment analysis was then performed to identify biological processes (BP) in which iDEGs were mostly involved (Fig. 4C). The iDEGs were significantly enriched in the BP ontology term including positive regulation of genes involved in cell proliferation and invasion such as “positive regulation of MAPK cascade (GO:0043410, *KISS1*, *IGFBP4*, *TNFRSF11B*, *IL11*)” and “positive regulation of cytosolic calcium ion concentration (GO:0007204, *CXCR4*, *CD24*, *EDNRB*, *GJA1*)”, indicating that maspin-induced gene signature is a functionally important factor for the promotion of invasiveness in MB-231-maspin cells.

### Possible role of the SRGN/TGFβ2 signaling axis in promoting EMT in MB-231-maspin cells

**Figure 5:**
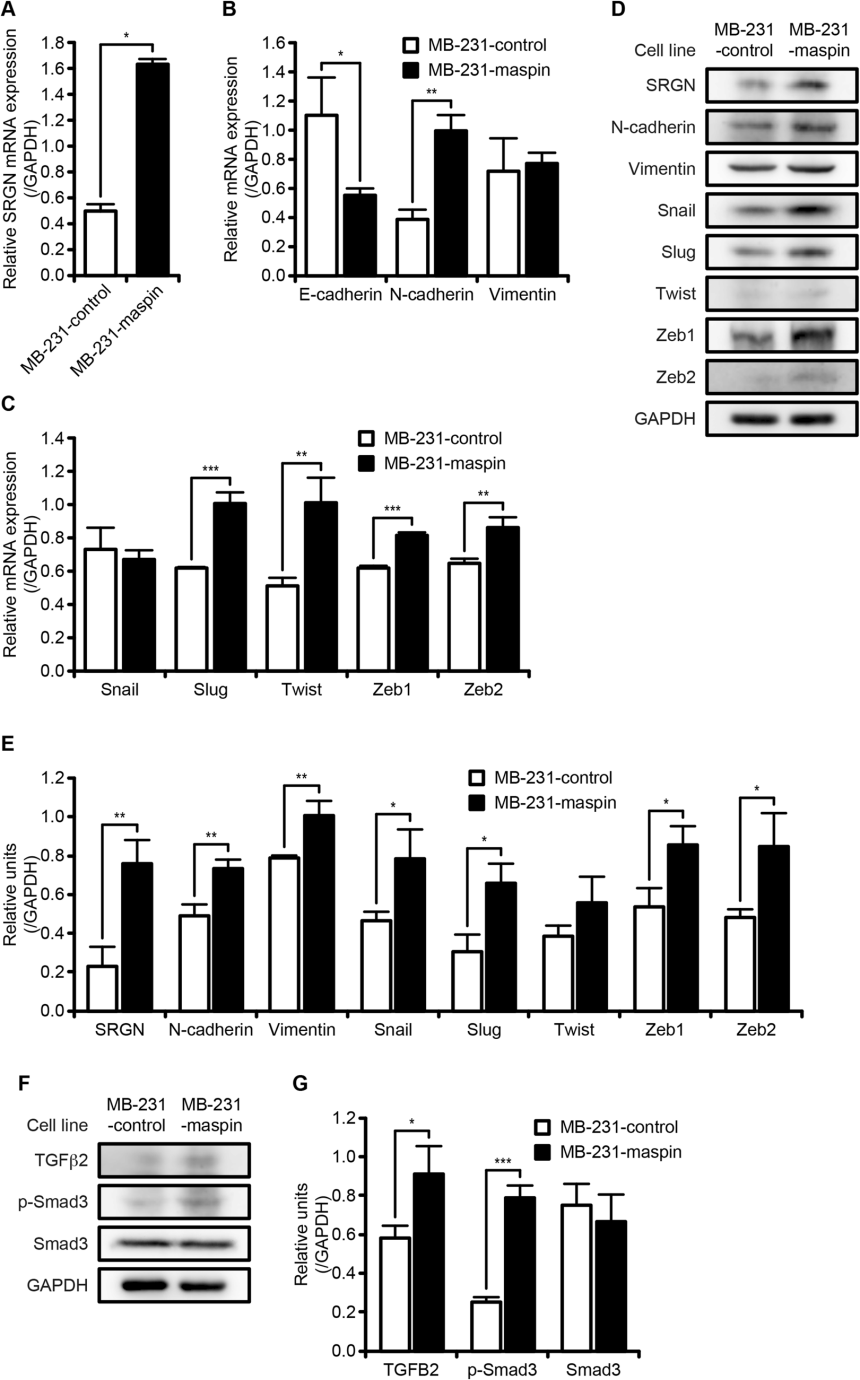
The protein expression of signaling factors involved in EMT. (**A**) Relative mRNA expression level of *SRGN*. mRNA expression was normalized to level of *GAPDH*. Data are shown as the mean ± SD (n = 3). *P < 0.05; Student’s *t*-test. (**B**) Relative mRNA expression levels of epithelial cell and mesenchymal cell marker. mRNA expression was normalized to level of *GAPDH*. Data are shown as the mean ± SD (n = 3). *P < 0.05, **P < 0.01; Student’s *t*-test. (**C**) Relative mRNA expression levels of EMT-related transcription factor. mRNA expression was normalized to level of GAPDH. Data are shown as the mean ± SD (n = 3). **P < 0.01, ***P < 0.001; Student’s *t*-test. (**D**) Protein expression levels of EMT-related markers. Expression level of GAPDH was used as a protein loading control. Whole western blots are presented in Supplementary Fig. S11. (**E**) Bar graph shows western blot quantification of SRGN, N-cadherin, Vimentin, Snail, Slug, Twist, Zeb1, and Zeb2. Densitometry analysis of each protein band was normalized to level of GAPDH. Data are shown as the mean ± SD (*n* = 3). *P < 0.05, **P < 0.01; Student’s *t*-test. (**F**) Protein expression levels of TGFβ signaling pathway factors. Expression level of GAPDH was used as a protein loading control. Whole western blots are presented in Supplementary Fig. S12. (**G**) Bar graph shows western blot quantification of TGFB2, phosphorylated Smad3, and Smad3. Densitometry analysis of each protein band was normalized to level of GAPDH. Data are shown as the mean ± SD (*n* = 3). *P < 0.05, ***P < 0.001; Student’s *t*-test.

On the basis of the microarray data, we used RT-qPCR to analyze the mRNA expression of *SRGN*, which is also known as a small molecule glycoprotein. In MB-231-maspin cells, *SRGN* mRNA level was significantly increased compared with that in MB-231-control cells (Fig. 5A). It has been reported that SRGN expression is involved in epithelial mesenchymal transition (EMT)^26,27^, prompting us to investigate the association between EMT and maspin in MDA-MB-231 cells. The mRNA level of E-cadherin, which is a well-known epithelial marker that is down-regulated during EMT, was significantly decreased in MB-231-maspin cells while mRNA level for the mesenchymal marker, N-cadherin, was significantly increased (Fig. 5B). Since it is well-known that EMT activation is regulated by the expression of several transcription factors, including *Snail*, *Slug*, *Twist*, *Zeb1*, and *Zeb2*, we performed RT-qPCR and western blot analysis for these transcription factors. The mRNA expression levels of *Slug*, *Twist*, *Zeb1*, and *Zeb2*, but not *Snail*, in MD-231-maspin cells were significantly higher than those in MB-231-control cells (Fig. 5C). It should be noted that no significant changes in the expression of these genes were found between HT-control and HT-maspin in MCF10A and MCF-7 cells (Supplementary Fig. S6), indicating that the changes in these gene expressions by maspin are specific to MDA-MB-231 cells. Moreover, SRGN, N-cadherin, and vimentin protein levels were significantly increased in MB-231-maspin cells (Fig. 5D,E). We also observed significantly increased protein expression levels of Snail, Slug, Zeb1, and Zeb2 protein in MB-231-maspin cells (Fig. 5D,E). Note that the protein level for Twist was not changed. It has been reported that SRGN enhances the invasive and metastatic potential in TNBC through a process consistent with EMT followed by increased expression of TGFβ2^28^. In agreement with this report, the expression of TGFβ2 protein in MB-231-maspin cells was higher compared with that in MB-231-control cells (Fig. 5F,G). Furthermore, Smad3 phosphorylated on Ser423/425, which serves as a reliable readout of TGFβ signaling pathway activity, was consistently increased in MB-231-maspin cells (Fig. 5F,G), indicating that cytoplasmic maspin enhances EMT in MDA-MB-231 cells via SRGN/TGFβ2 signaling axis.

## Discussion

According to the previous reports, maspin is presumed to act either as a tumor suppressor or as an oncogene. The anti-tumor mechanisms of maspin function are well studied because maspin has long been considered as a tumor suppressor gene. For example, maspin overexpression in tumor cells was shown to induce apoptosis through the enhanced release of cytochrome c or via upregulation of Bax, a well-known pro-apoptotic protein^9,29^. Moreover, maspin protein is able to directly bind to histone deacetylase C1 (HDAC1) and inhibit its activity that regulates histone deacetylation to suppress transcription of several genes. In prostate cancer cell lines, re-expression of maspin reportedly increased the expression of tumor suppressor genes that are under the control of HDAC1, such as CK18, p21^waf1/CIP1^, and Bax^30^. However, the molecular mechanisms underlying maspin-mediated tumor progression remain unclear. To our knowledge, this study is the first to show overexpression of cytoplasmic maspin promotes the cell invasion capability in MDA-MB-231 cells (Fig. 3A,B). On the other hand, the promotion of cell invasion was not observed in MCF10A and MCF-7 cells, both of which are also known as non-invasive cell lines (Fig. 3A,B). These results are in agreement with our previous papers that maspin is frequently overexpressed in TNBC, which is known to have an aggressive clinical behavior^31^. These observations indicate that overexpression of cytoplasmic maspin does not give rises to invasive behavior in breast cancer cells with non-aggressive phenotype, but it potentiates invasive capability in some aggressive breast cancer cells such as TNBC cells.

GO term enrichment analysis is widely used for exploring the biological significance of the alterations of gene expression levels^32^. Our analysis showed that *KISS1*, *IGFBP4*, *TNFRSF11B*, and *IL11*, which are annotated to the GO term “positive regulation of MAPK cascade”, were significantly upregulated in MB-231-maspin cells (Fig. 4B,C). It has been reported that MAPK signaling cascade plays a crucial role in the development and progression of various cancers by regulating cell proliferation, drug resistance, apoptosis, angiogenesis, cell migration, and metastasis^33^. Among four genes identified as iDEGs, *KISS1*, which encodes an endogenous ligand that binds to KISS1R (also referred to as GPR54), was recently reported to contribute to TGFβ-mediated cell invasion of MDA-MB-231 cells, and its expression was reportedly upregulated in patients with TNBC^34,35^. Furthermore, it is demonstrated that siRNA-mediated inhibition of *TNFRSF11B* expression, known as the gene encoding osteoprotegerin and a secreted member of the tumor necrosis factor receptor superfamily, reduced the metastatic capability of TNBC cell lines in the chick embryo metastasis model^36^. We also found that SRGN expression level was higher in MB-231-maspin cells than that in the MB-231-control cells (Fig. 5A,D). SRGN has been characterized as a low molecular weight proteoglycan secreted from hematopoietic cells that interacts with several biological molecules including extracellular matrix components, proteolytic enzymes, chemokines, cytokines, and cell surface receptors^37^. Reports documenting that SRGN promotes cancer cell aggressiveness and metastasis through several signaling pathways, including CD44/NF-κB/CLDN1 axis, HIF-1α/SRGN axis, and SRGN/TGFβ2 axis, may further support our observations presented here^28,38,39^. Indeed, we demonstrated that SRGN, TGFβ2, phosphorylated Smad3, and EMT-related transcription factors, including Snail, Slug, Zeb1, and Zeb2, had a higher level of expression in MB-231-maspin cells as compared with that in MB-231-control cells (Fig. 5D,E). These results raise a strong possibility that the increased level of expression of the cytoplasmic maspin in MDA-MB-231 cells promotes cell invasion by facilitating EMT through SRGN/TGFβ2 signaling axis. Although detailed molecular mechanisms by which cytoplasmic maspin activates SRGN/TGFβ2 axis remain to be clarified, our data suggest that cytoplasmic maspin plays a pivotal role in the aggressive phenotype and serves as a therapeutic target in patients with breast cancer, especially TNBC.

In cancer development and progression, some genes display both oncogenic and tumor-suppressive functions depending on the cellular context and subcellular localization^40^. Besson et al*.* reported that mislocalization of p27^kip1^, a well-known tumor suppressor that inhibits cell cycle progression by regulating cyclin/cyclin-dependent kinase (CDK) complexes, to the cytoplasm of tumor cells induces cyclin/CDK-independent oncogenic activity in cancer cells^41^. In accordance with these reports and our findings, it is presumed that biological functions of maspin in cancer cells are regulated through its subcellular localization. Therefore, understanding the molecular mechanisms underlying nuclear translocation process of maspin is important for improving the prognosis of patients with breast cancer. We observed that exogenous expression of maspin cDNA generated from MCF10A showed cytoplasmic localization in MDA-MB-231, whereas its expression showed pan-cellular localization in MCF10A (Fig. 2A–C), suggesting that the nuclear translocation of maspin in breast cancer cells depends on the carrier, but not tag sequence including NLS. Additionally, the fact that NSL-fused maspin was expressed in both the nucleus and cytoplasm in breast cancer cell lines (Supplementary Fig. S4A–C) suggests that importin-dependent classical nuclear translocation mechanism works normally in these cells and that another unknown pathway is involved in the nuclear translocation of maspin. In accordance with our findings, Dzinic et al*.* observed that leptomycin B, a specific inhibitor of nuclear export protein CRM1, significantly reduced nuclear accumulation of maspin, but not that of the mutant maspin (maspin^D346E^), and speculated that subcellular localization of maspin may be indirectly controlled by an unknown factor through a CRM1-dependent export mechanism^42^. Alternatively, Reina et al*.* reported that the sequence of 28 amino acids in maspin protein (KLIKRLYVDKSLNLSTEFI-SSTKRPYAK) acts as a NLS and speculated that the nuclear-cytoplasmic transport of maspin may be controlled through the regulation of its three-dimensional conformation^43^. Although these reports help us in understanding the mechanisms underlying subcellular translocation of maspin, the exact details of this process remain unclear. Therefore, further studies are needed to elucidate the molecular mechanisms that control subcellular localization of maspin, especially shifting from oncogenic to tumor-suppressive or vice versa.

We demonstrated that cytoplasmic maspin plays an important role in promoting the invasion of MDA-MB-231 cells and induces the expression of several genes enriched in the GO term involving the positive regulation of cell proliferation and invasion. Moreover, MDA-MB-231 cells that stably express maspin show elevated expression of SRGN, Snail, Slug, Zeb1, and Zeb2, as well as the activation of TGFβ signaling pathway, suggesting that cytoplasmic maspin promotes EMT through activation of SRGN/TGFβ2 signaling axis. Taken together, these findings suggest that cytoplasmic maspin might be a potential therapeutic target. In particular, the regulation of cytosol-to-nuclear translocation of maspin may serve as a novel therapeutic approach to improve the prognosis of patients with aggressive breast cancer.

## Material and methods

### Cell culture

Human breast cancer cell lines, MCF-7 and MDA-MB-453, were provided by the RIKEN BioResource Center (BRC) through the National Bio-Resource Project of the MEXT, Japan. Human breast cancer cell line, MDA-MB-231, and human mammary epithelial cell line, MCF10A, were purchased from the American Type Culture Collection (ATCC). MCF-7 cells were maintained in Dulbecco’s Modified Eagle Medium (DMEM; Nissui Pharmaceutical; Tokyo, Japan) supplemented with 10% inactivated fetal bovine serum (FBS; Biological Industries, Cromwell, CT, USA) in humidified atmosphere of 5% CO_2_ at 37 °C. MDA-MB-453 and MDA-MB-231 cells were maintained in Leibovitz-L15 medium (Sigma-Aldrich, St. Louis, MO, USA) supplemented with 10% inactivated FBS in humidified atmosphere of 0% CO_2_ at 37 °C. MCF10A cells were grown in mammary epithelial basal medium (MEBM; Lonza, Walkersville, MD, USA) supplemented with BPE, hydrocortisone, hEGF, insulin, gentamicin/amphotericin-B, and 100 ng/ml cholera toxin in humidified atmosphere of 5% CO_2_ at 37 °C. 293FT cell line was purchased from Thermo Fisher Scientific (Waltham, MA, USA) and was maintained in DMEM supplemented with 10% inactivated FBS in humidified atmosphere of 5% CO_2_ at 37 °C.

### Plasmid and siRNA transfection

MCF10A, MCF7, and MDA-MB-231 cells were seeded into 6-well plate at a density of 3.6 × 10^5^ cells/well and were allowed to adhere overnight. For each well, cells were transiently transfected with 2.5 μg/well of plasmid using 7.5 μL of FuGENE HD (Promega, Madison, WI, USA) according to the manufacturer’s instructions. Ten nanomolar maspin siRNA (Silencer Select siRNA, s10468, Thermo Fisher Scientific) or control siRNA (Silencer Negative Control #1, Thermo Fisher Scientific) was transiently transfected with 9 μL Lipofectamine RNAiMAX (Thermo Fisher Scientific) according to the manufacturer’s instructions.

### RT-qPCR

Total RNA from cells was recovered using TRIzol Reagent (Thermo Fisher Scientific). Isolated RNA was reverse-transcribed using High-Capacity RNA-to-cDNA Kit (Thermo Fisher Scientific) according to the manufacturer’s instructions. Gene expression levels were determined by TaqMan Gene Expression Master Mix (Thermo Fisher Scientific) using LightCycler 96 system (Roche Diagnostics, Mannheim, Germany). Primer and probe sets used in this study were summarized in Supplementary Table S2. Relative mRNA expression levels were normalized by *GAPDH* as an internal reference gene.

### Western blot

Whole cells were lysed in RIPA buffer (50 mM Tris–HCl [pH 8.0], 150 mM NaCl, 1% NP-40, 0.5% sodium deoxycholate, 0.1% sodium dodecyl sulfate [SDS]) supplemented with protease inhibitor cocktail (Roche Diagnostics) and phosphatase inhibitor cocktail (Roche Diagnostics). In nuclear/cytoplasmic fractionation assay, nuclear and cytoplasmic fraction were extracted using subcellular protein fractionation kit for cultured cells (Thermo Fisher Scientific) according to the manufacturer’s instructions. The determination of protein concentration was performed by the Bradford protein assay (Bio-Rad Laboratories, Hercules, CA, USA), and then protein concentration was adjusted with 5 × sample buffer (60 mM Tris–HCl (pH6.8), 5% β-mercaptoethanol, 0.1% bromophenol blue, 2% SDS, 25% glycerol) and RIPA buffer. Samples were boiled for 5 min, then SDS–polyacrylamide gel electrophoresis (SDS-PAGE) and transferred onto the 0.45 μm PVDF membrane (Merck Millipore, Darmstadt, Germany) were performed. Membranes were cut prior to blocking with ECL Prime blocking reagent (GE Healthcare, Buckinghamshire, UK), and then immunoblotting was performed. The primary antibodies used in this experiment were listed in Supplementary Table S3. The protein bands were detected by ImageQuant LAS4000 (GE healthcare). Quantification of protein bands from western blot was performed using ImageJ/Fiji software (ver. 2.1.0/1.53c).

### Immunofluorescence

Cells were plated in a Nunc Lab-Tek II 8-well chamber slide (Thermo Fisher Scientific) and were allowed to adhere overnight. Cells were washed two times with phosphate-buffer saline (PBS) and fixed with 4% paraformaldehyde for 15 min at room temperature (RT). Fixed cells were washed with PBS and permeabilized with 100% ice-cold methanol for 10 min at − 20 °C. Samples were treated with 0.2% Triton-X in PBS for 10 min at RT. After blocking with 10% goat serum in PBS for 60 min at RT, samples were incubated with anti-maspin antibody (clone G167-70, 554292, BD Pharmingen, San Diego, CA, USA and clone EAW24, NCL-MASPIN, Leica Biosystems, Newcastle Ltd., UK) at 4 °C overnight, followed by incubation with Alexa Fluor 488-conjugated secondary antibody (Thermo Fisher Scientific). Cells transfected with the plasmid DNA expressing HaloTag-fused maspin were labeled with TMRDirect ligand (Promega) according to the manufacturer’s instructions. Slides were mounted by ProLong Diamond Antifade Mountant containing DAPI (Thermo Fisher Scientific). Confocal fluorescent images were obtained using confocal laser canning microscopy (FV1000D, Olympus, Tokyo, Japan).

### Cell proliferation assay

Cells transfected with either plasmid DNA or siRNA were plated in a 96-well plate (TPP, Trasadingen, Switzerland) and incubated for the time ranging between 24 and 72 h. Cell proliferation was determined using Cell Counting Kit-8 (Dojin Kagaku, Kumamoto, Japan) according to the manufacturer’s instruction. Absorbance at 450 nm and 600 nm wavelength was measured by the plate reader.

### Cell invasion assay

Cells transfected with plasmid DNA or siRNA were starved in serum-free medium for 18 h and then seeded into upper chamber of transwells with 8.0 μm pore size membrane in 24-well plate. The lower chambers were filled with a medium containing 10% inactivated FBS. After 48 h of incubation, QCM ECMatrix Cell Invasion Assay, 24-well (8.0 μm), fluorimetric kit (Merck Millipore, Billerica, MA, USA) was used to measure the cell invasion capability according to the manufacturer’s instructions. Fluorescence intensity was measured by the fluorescence microplate reader.

### Plasmid DNA preparation and lentivirus production

To generate the plasmid DNA expressing HaloTag-fused maspin (pHT-maspin), a full length maspin cDNA was amplified from MCF10A cells by PCR with a primer set (HT-maspin cln SgfI F, HT-maspin cln PmeI R) and ligated into pFN21A vector (Promega) according to the manufacturer’s instructions. Similarly, plasmid DNA expressing HaloTag and SV40 large T antigen derived NLS (amino acid sequence: PKKKRKV) was also constructed based on the pFN21A vector (a primer set: HT-maspin cln SgfI F, HT-maspin-NLS cln PmeI R).

For the production of a lentiviral vector capable of expressing maspin, maspin cDNA amplified from MCF10A cells by PCR with a primer set (Maspin cln HindIII F, Maspin cln NheI R) and ZsGreen cDNA, which encodes green fluorescent protein, from pZsGreen1-N1 (Takara Bio inc., Shiga, Japan) by PCR with a primer set (ZsGreen cln BsaI F, ZsGreen cln BsaI R) were linked by a P2A self-cleaving peptide (P2A-BsaI F, P2A-BsaI R) and then subcloned into pX333 vector (Addgene, #64073, Cambridge, MA, USA). Maspin-P2A-ZsGreen sequence and ZsGreen were subsequently amplified with an attB adapter primer set (attB1-maspin-ZsGreen F, attB1-ZsGreen F, attB2-maspin-ZsGreen R). Two attB-flanked PCR products were transferred into pDONR221 Gateway entry vector (Thermo Fisher Scientific) by using a BP Clonase II Enzyme mix (Thermo Fisher Scientific) and subsequently transferred into pLenti6.4/R4R2/V5-DEST destination vector (Thermo Fisher Scientific) with pENTR5/CMVp (Thermo Fisher Scientific) by using LR clonase II Plus Enzyme mix (Thermo Fisher Scientific) to generate lentivirus expression vector (pLenti/maspin-ZsGreen, pLenti/ZsGreen). pLenti/maspin-ZsGreen and pLenti/ZsGreen vectors were co-transfected with ViraPower Packaging Mix (Thermo Fisher Scientific) into 293FT cells, and lentivirus stocks were collected according to the manufacturer’s instructions. Primers and oligonucleotides used in plasmid DNA construction are listed in Supplementary Table S4.

### Establishment of MDA-MB-231 cell line for stable maspin overexpression

To establish MDA-MB-231 stable cell lines that overexpress either maspin-ZsGreen or ZsGreen alone (control), cells were infected with lentivirus-based vector and selected for lentiviral integration with 10 μg/mL blasticidin (Fujifilm Wako Pure Chemical Corporation, Osaka, Japan). The surviving clones were pooled to establish MB-231-maspin and MB-231-control cell lines.

### DNA microarray analysis

Total RNA from MB-231-maspin and MB-231-control cells was recovered using RNeasy Mini Kit (QIAGEN, Valencia, CA, USA) according to the manufacturer’s instructions. For DNA microarray analysis, isolated RNA was labeled using Cy5 and hybridized onto the 3D-Gene human mRNA oligo chip 25 k Ver 2.1 (Toray, Kamakura, Japan). The signal intensities of each gene were detected using 3D-Gene Scanner 3000 (Toray) and subtracted with the background mean and normalized to the value in which the median intensity was equal to 25. The dataset generated by gene expression microarray have been deposited in the NCBI Gene Expression Omnibus (GEO) repository (GSE144044). Gene expression profiles in which the mean of normalization values in MB-231-control or MB-231-maspin were higher than 10 were used for the analysis of DEGs using Excel (Microsoft Corporation, Redmond, WA, USA) and R studio (Version 1.2.5001). GO term enrichment analysis was performed with DAVID online tool.

### Statistical analysis

All experimental values were presented as mean ± standard deviation (SD). Data calculation was performed with Excel and R studio. Student *t* test was used for statistical comparison between two data sets. *P*-values less than 0.05 were considered to be statistically significant.

## Supplementary Information

Supplementary Figures.

Supplementary Figures.

Supplementary Table S1.

Supplementary Table S2.

Supplementary Table S3.

Supplementary Table S4.
