# Supplementary material for: Role of cytoplasmic localization of maspin in promoting cell invasion in breast cancer with aggressive phenotype
Source: Sci Rep. 2021 May 31;11:11321. doi: 10.1038/s41598-021-90887-z (PMC8166868; doi:10.1038/s41598-021-90887-z)
Supplement: Supplementary file 1 — Supplementary Figures. [file 41598_2021_90887_MOESM1_ESM.pdf]

Figure S1

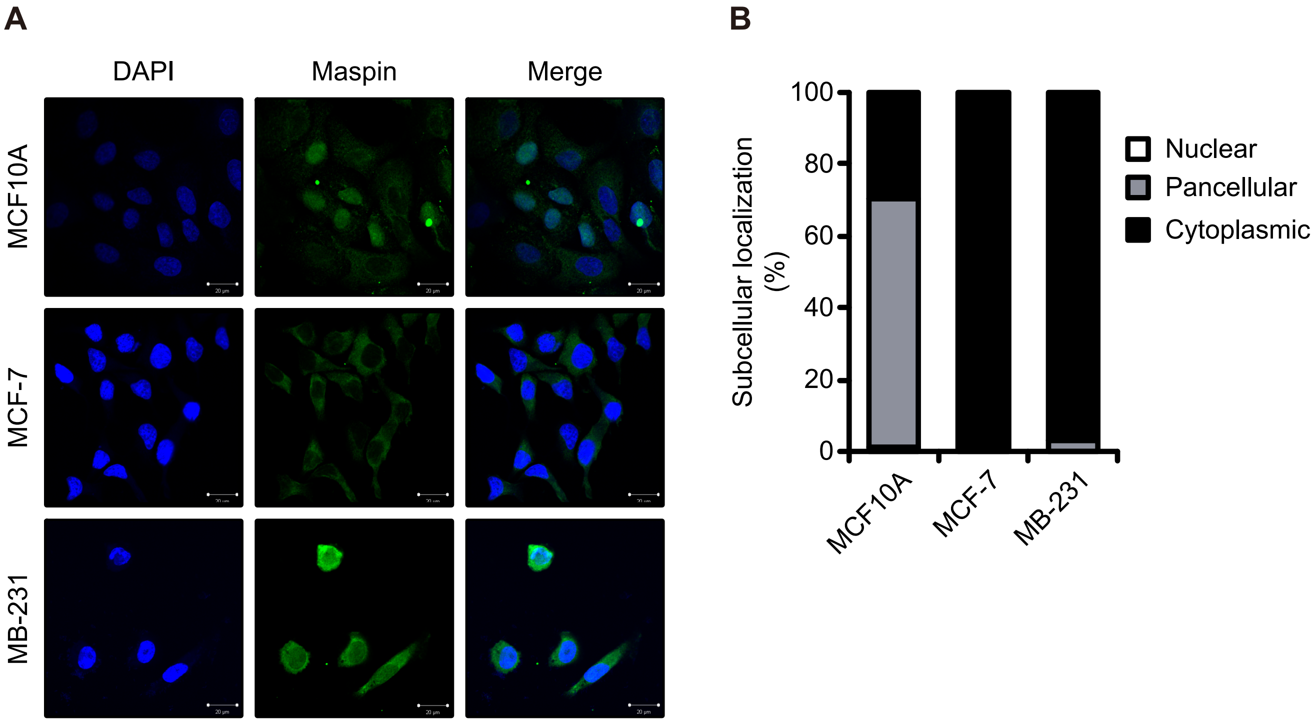

Figure S1 Subcellular localization of maspin protein in MCF10A, MCF-7, and MDA-MB-231 cells. (A) Representative images of subcellular localization of maspin protein in MCF10A, MCF-7, and MDA-MB-231 cells. Maspin protein was detected using anti-maspin antibody purchased from Leica Biosystems (clone EAW24). Nuclei was counterstained with DAPI. Scale bars represent 20 μm. Images were acquired using Zeiss LSM 780 Systems confocal laser scanning microscope and Zeiss ZEN2011 software (ver. 11.0.4.190). (B) Quantification of the subcellular localization of maspin. Bar graph shows the percentage of maspin distribution that is calculated by counting number of cells exhibiting pancellular, nuclear, or cytoplasmic distribution (n = 5).

Figure S2

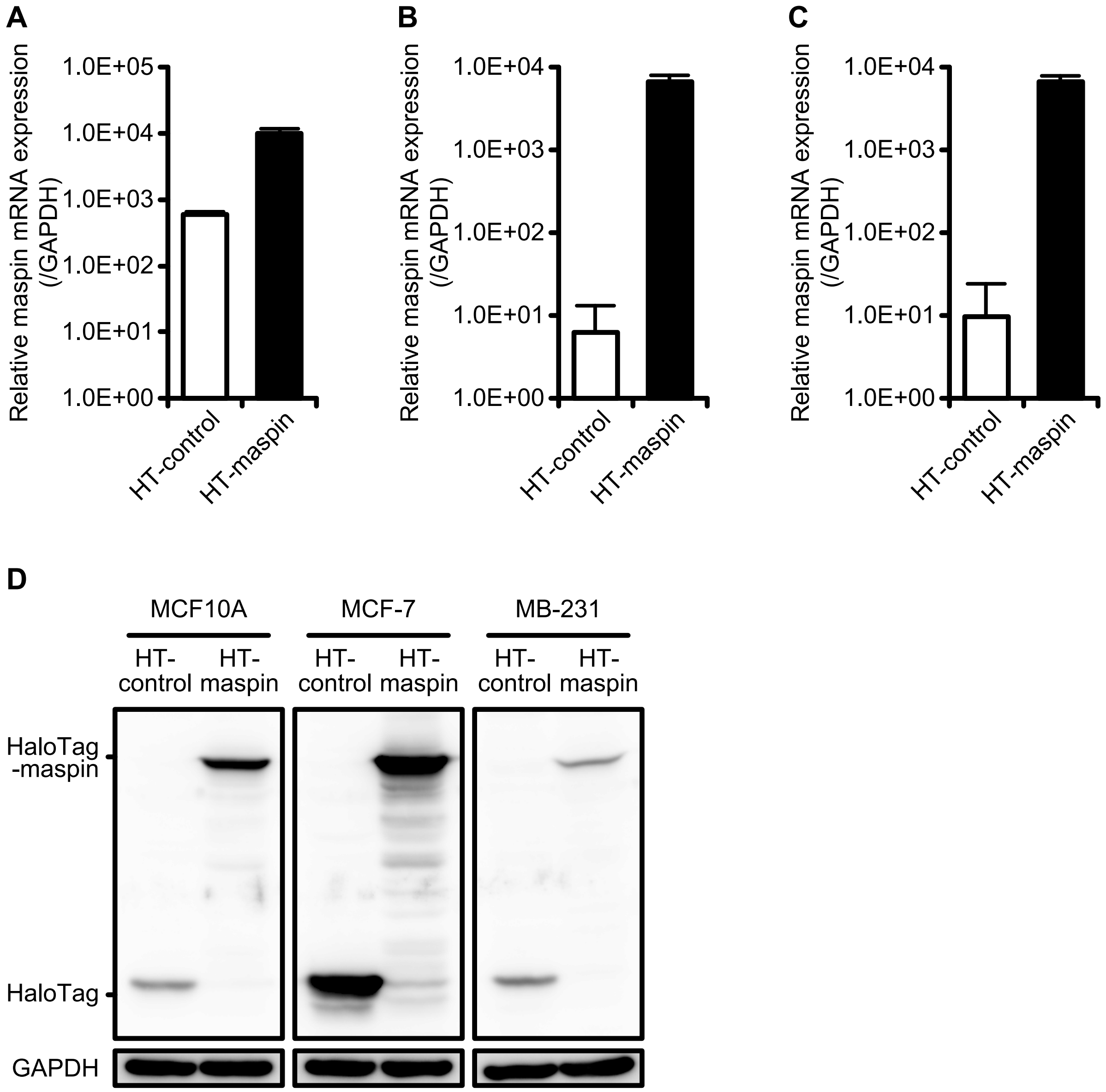

Figure S2 Expression levels of maspin mRNA and protein. **(A, B, C)** Overexpression of HaloTag-fused maspin or HaloTag control in MCF10A **(A)**, MCF-7 **(B)**, and MDA-MB-231 **(C)** cells. mRNA expression was normalized to level of GAPDH. Data is shown as a mean ± SD (n = 3). **(D)** Protein expression of HaloTag-fused maspin. Expression level of GAPDH was used as a protein loading control. Whole western blots are presented in Supplementary Fig S13.

Figure S3

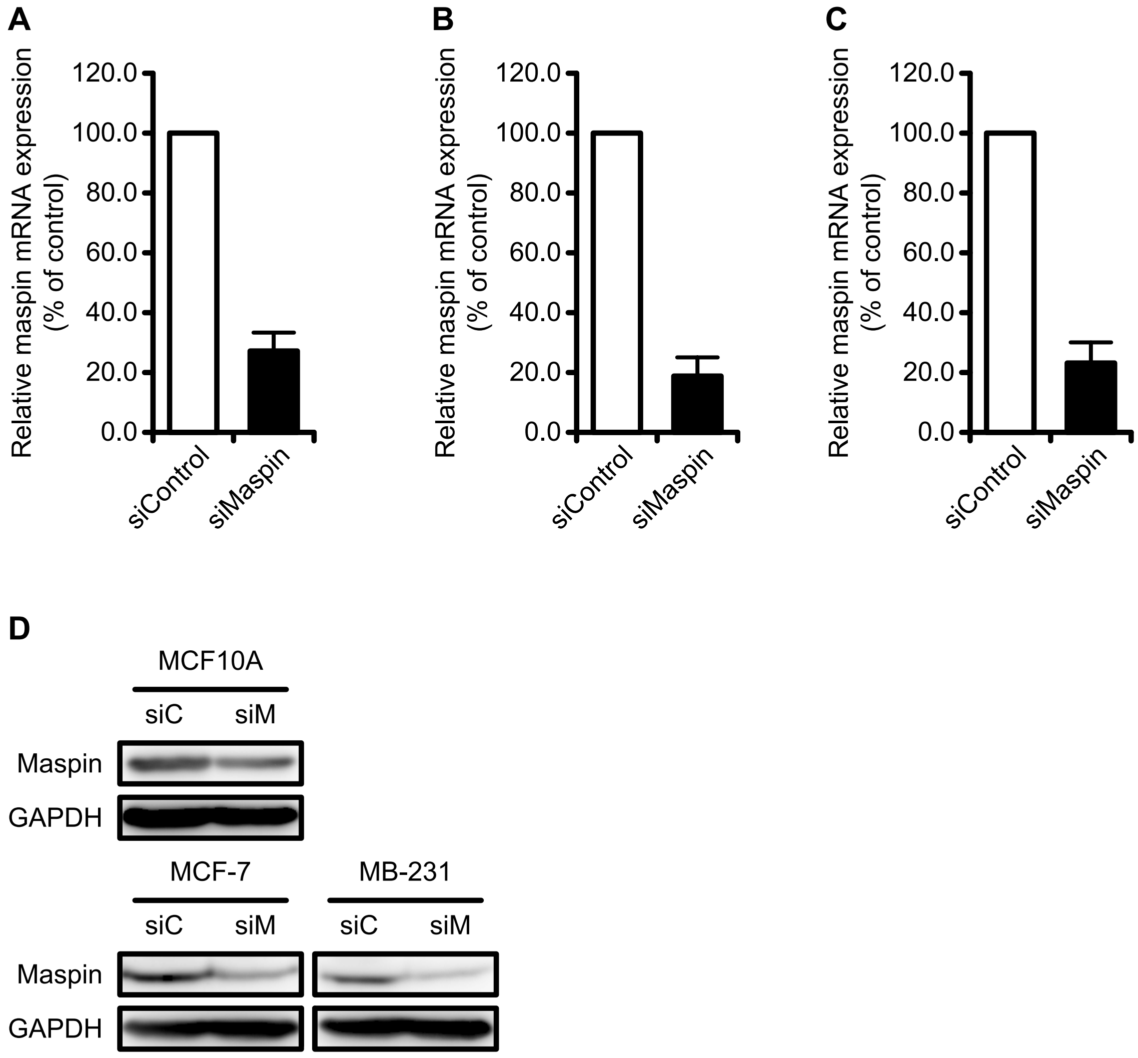

Figure S3 Expression levels of maspin mRNA and protein. **(A, B, C)** Downregulation of maspin in MCF10A **(A)**, MCF-7 **(B)**, and MDA-MB-231 **(C)** cells. mRNA expression was normalized to level of GAPDH. Data is shown as a mean ± SD (n = 3). **(D)** Protein expression level of maspin in cells expressing maspin siRNA or control siRNA. GAPDH is shown for loading control. Whole western blots are presented in Supplementary Fig S14.

Figure S4

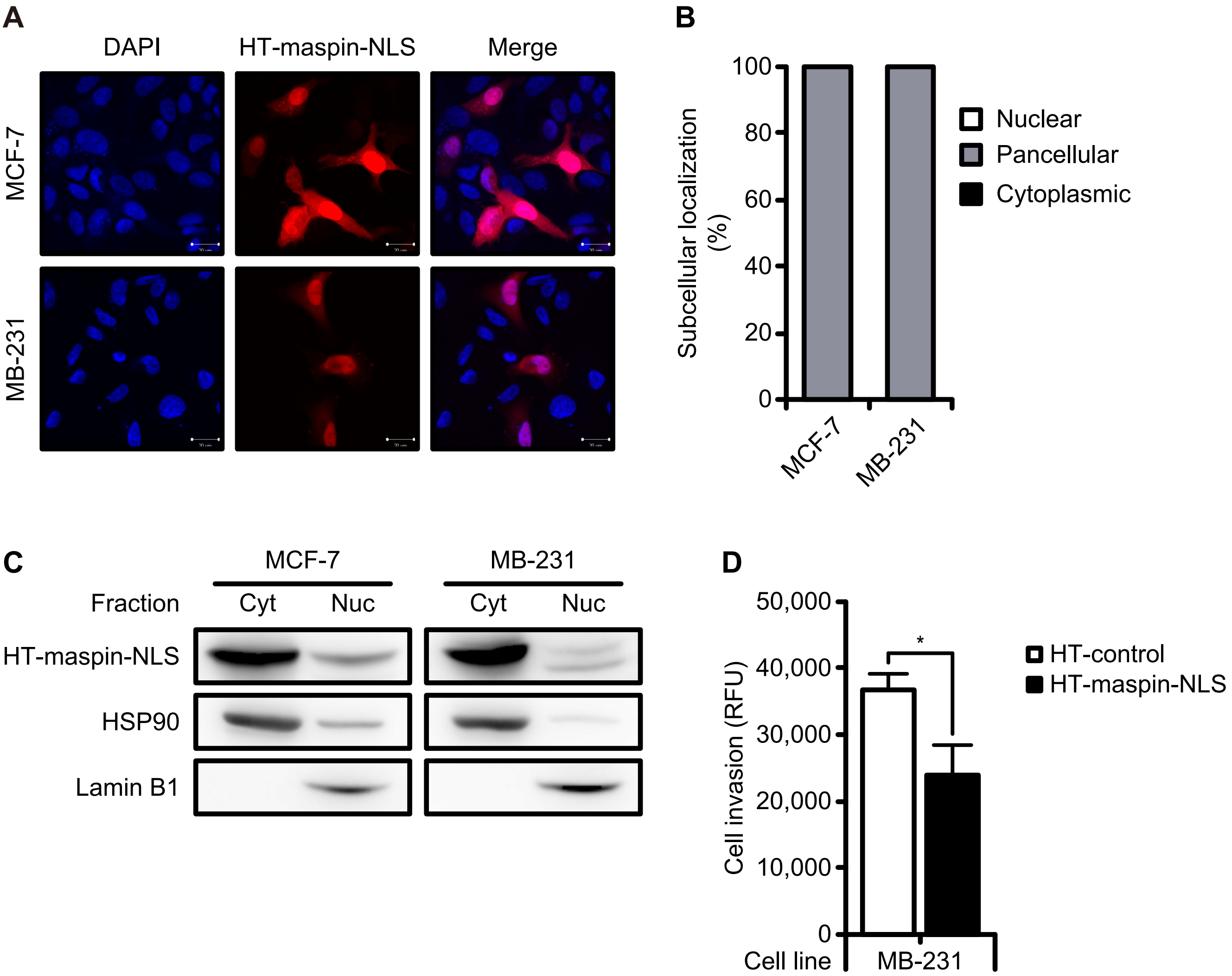

Figure S4 The relevance of HaloTag and NLS-fused maspin subcellular localization to cell invasion in MCF-7 and MDA-MB-231 cells. **(A)** Representative images of subcellular localization of HaloTag and NLS-fused maspin. Nuclei was counterstained with DAPI. Scale bars represent 20 μm. Images were acquired using Zeiss LSM 780 Systems confocal laser scanning microscope and Zeiss ZEN2011 software (ver. 11.0.4.190). **(B)** Quantification of the subcellular localization of HaloTag and NLS-fused maspin. Bar graph shows the percentage of maspin distribution that is calculated by counting number of cells exhibiting pancellular, nuclear, or cytoplasmic distribution (n = 5). **(C)** Protein expression of HaloTag and NLS-fused maspin in the cytoplasmic (Cyt) and nuclear (Nuc) fractions. Expression levels of HSP90 and Lamin B1 was used as protein loading control in the cytoplasmic and nuclear fractions, respectively. Whole western blots are presented in Supplementary Fig S15. **(D)** Cell invasion capability of MDA-MB-231 cells overexpressing HaloTag and NLS-fused maspin or HaloTag-control. Data are shown as the mean ± SD (n = 3). *P < 0.05; Student's t-test.

Figure S5

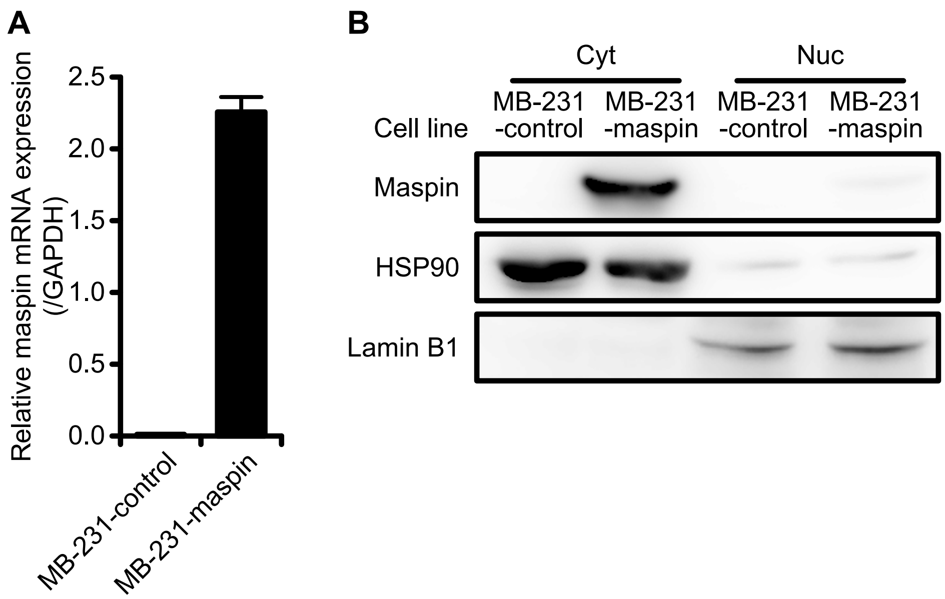

Figure S5 Confirmation of the maspin expression in MDA-MB-231 cells stably expression maspin. **(A)** Relative mRNA expression levels of maspin. mRNA expression was normalized to level of GAPDH. Data is shown as a mean ± SD (n = 3). **(B)** Protein expression of maspin in the cytoplasmic (Cyt) and nuclear (Nuc) fractions from MB-231-control and MB-231-maspin cells. Expression levels of HSP90 and Lamin B1 was used as protein loading control in the Cyt and Nuc fractions, respectively. Whole western blots are presented in Supplementary Fig S16.

Figure S6

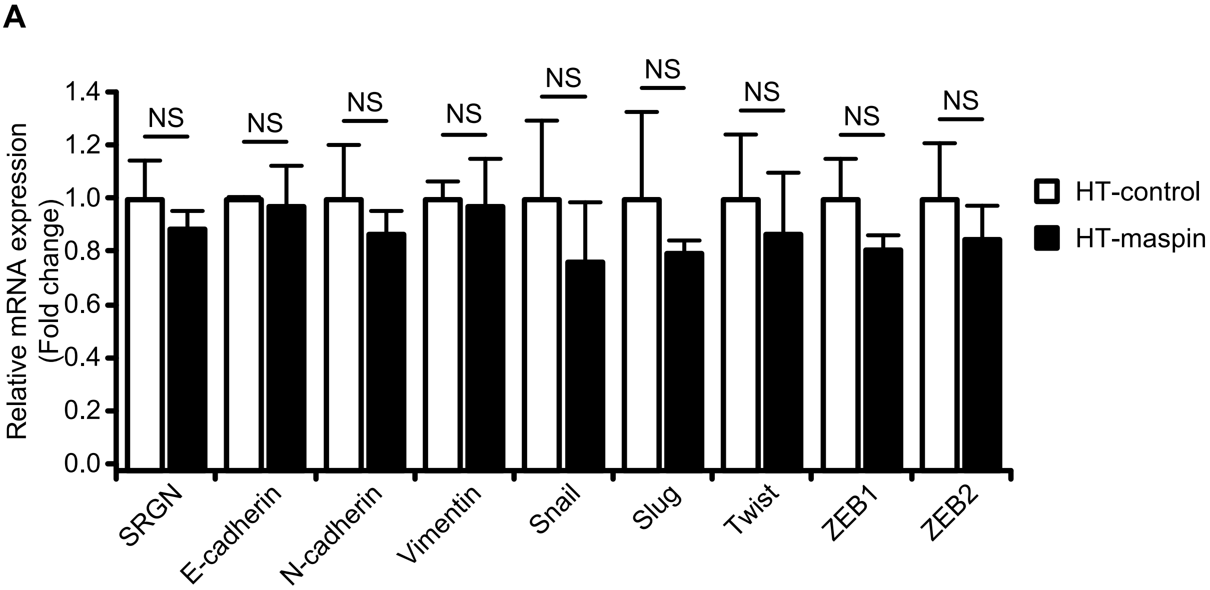

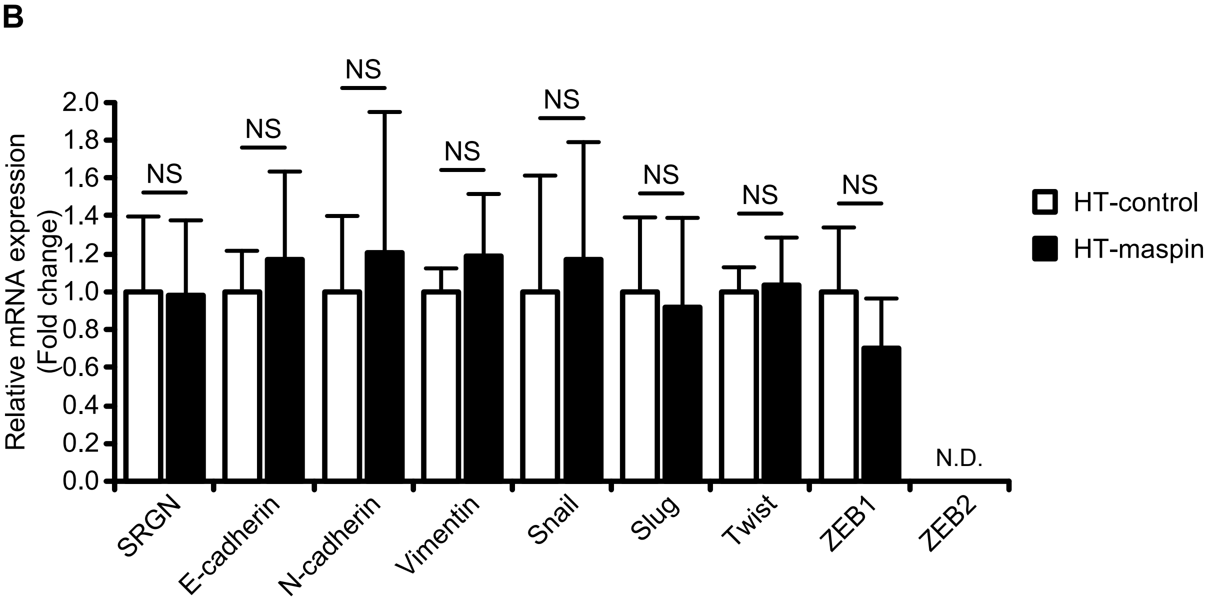

Figure S6 Relative mRNA expression levels of SRGN, epithelial cell marker, mesenchymal cell marker, and EMT-related transcription factors. Overexpression of HaloTag-fused maspin or HaloTag control in MCF10A (A) and MCF-7 (B). mRNA expression was normalized to level of GAPDH and represented as fold change relative to the expression in cells expressing HT-control. Data is shown as a mean ± SD (n = 3).
