# Supplementary material for: Role of cytoplasmic localization of maspin in promoting cell invasion in breast cancer with aggressive phenotype
Source: Sci Rep. 2021 May 31;11:11321. doi: 10.1038/s41598-021-90887-z (PMC8166868; doi:10.1038/s41598-021-90887-z)
Supplement: Supplementary file 2 — Supplementary Figures. [file 41598_2021_90887_MOESM2_ESM.pdf]

Figure S7

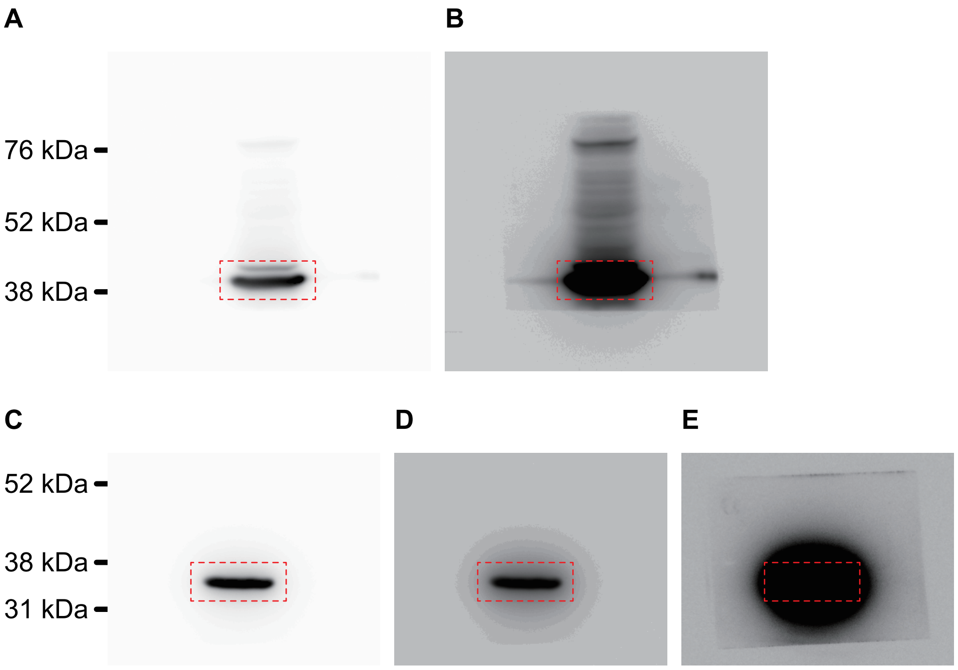

Figure S7 Whole western blots from Figure 1B in the manuscript. Amersham full-range rainbow molecularweight marker was used to calculate sample molecular weights and sizes in kDa are indicated. Panels A and C show the original images used for the detection of each protein. The brightness and contrast were not changed in the process of acquiring this images. Panels B, D, and E are the same images as A and C, but with different brightness, contrast, and exposure. Red dotted lines show the cropping locations.

Figure S8

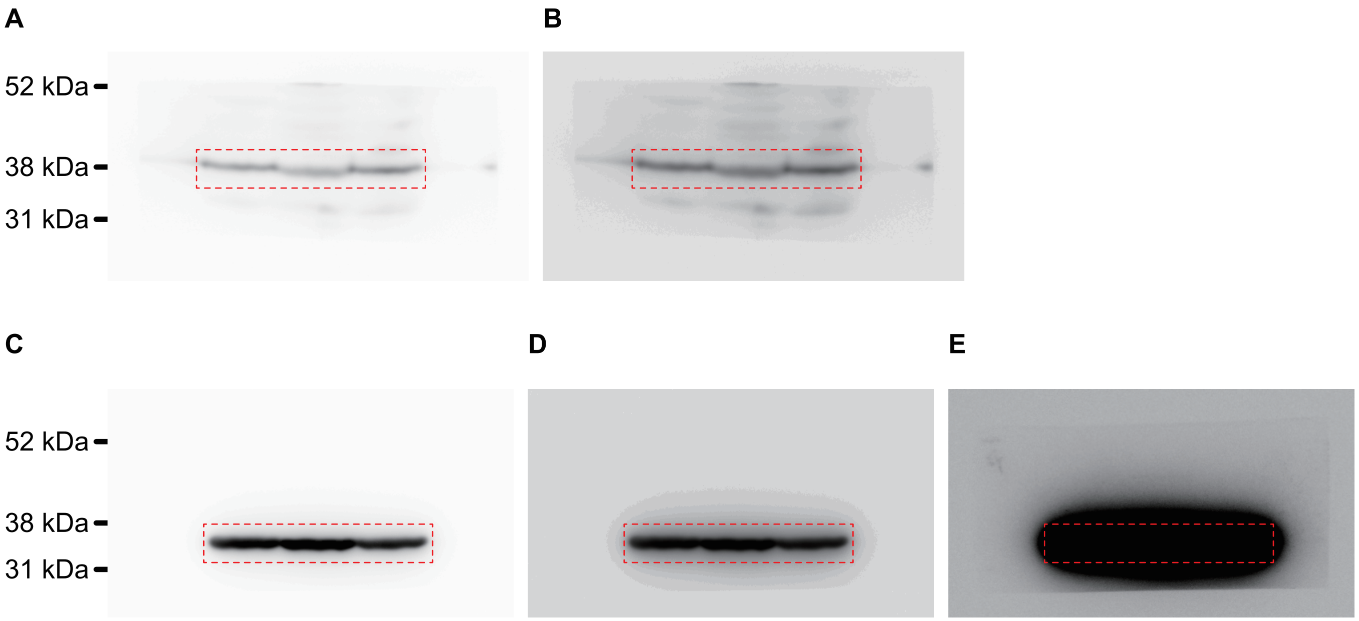

Figure S8 Whole western blots from Figure 1D in the manuscript. Amersham full-range rainbow molecularweight marker was used to calculate sample molecular weights and sizes in kDa are indicated. Panels A and C show the original images used for the detection of each protein. The brightness and contrast were not changed in the process of acquiring this images. Panels B, D, and E are the same images as A and C, but with different brightness, contrast, and exposure. Red dotted lines show the cropping locations.

Figure S9

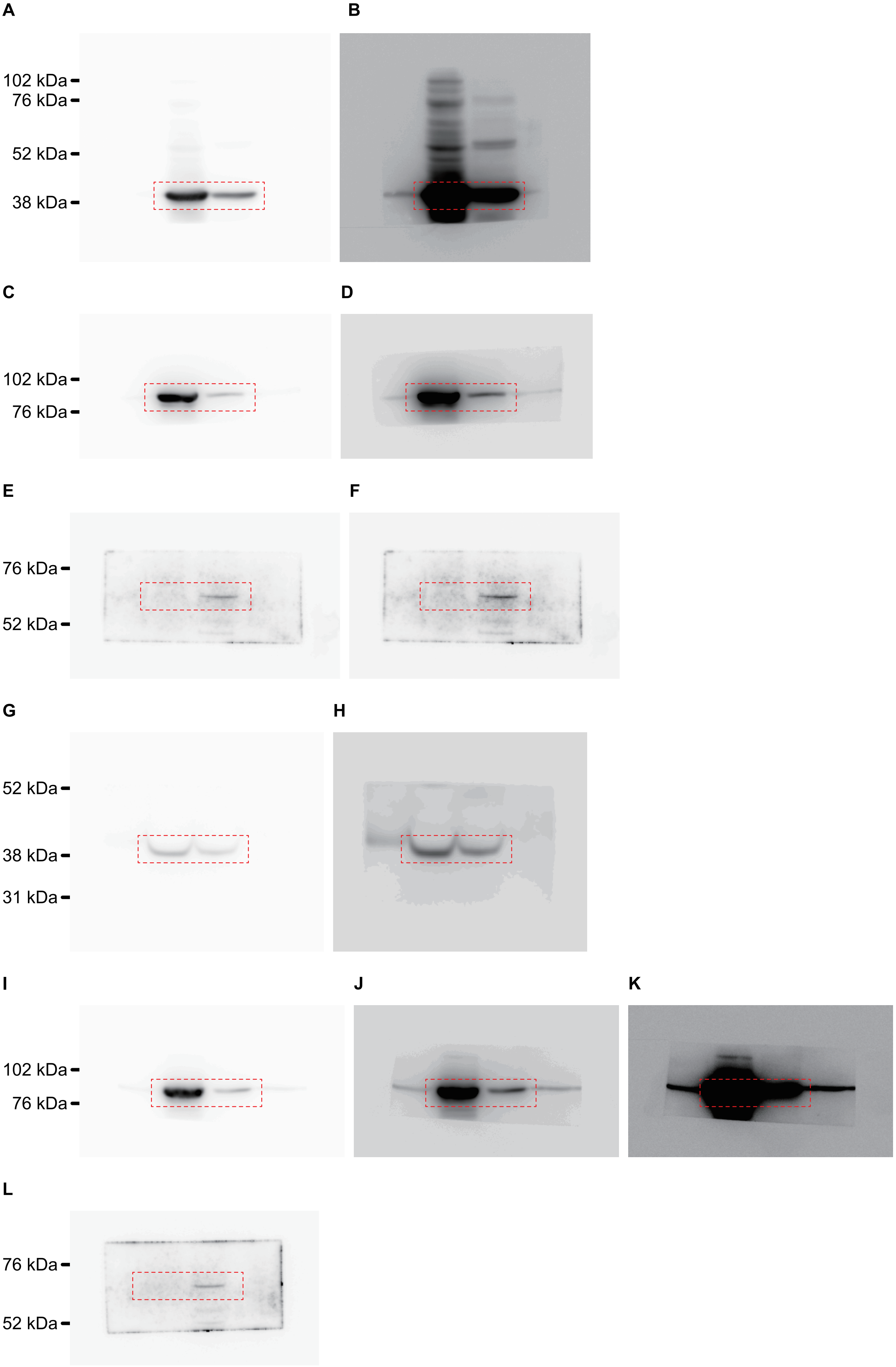
A
102 kDa
76 kDa
52 kDa
38 kDa
B
C
102 kDa
76 kDa
D
E
76 kDa
52 kDa
F
G
52 kDa
38 kDa
31 kDa
H
I
102 kDa
76 kDa
J
K
L
76 kDa
52 kDa

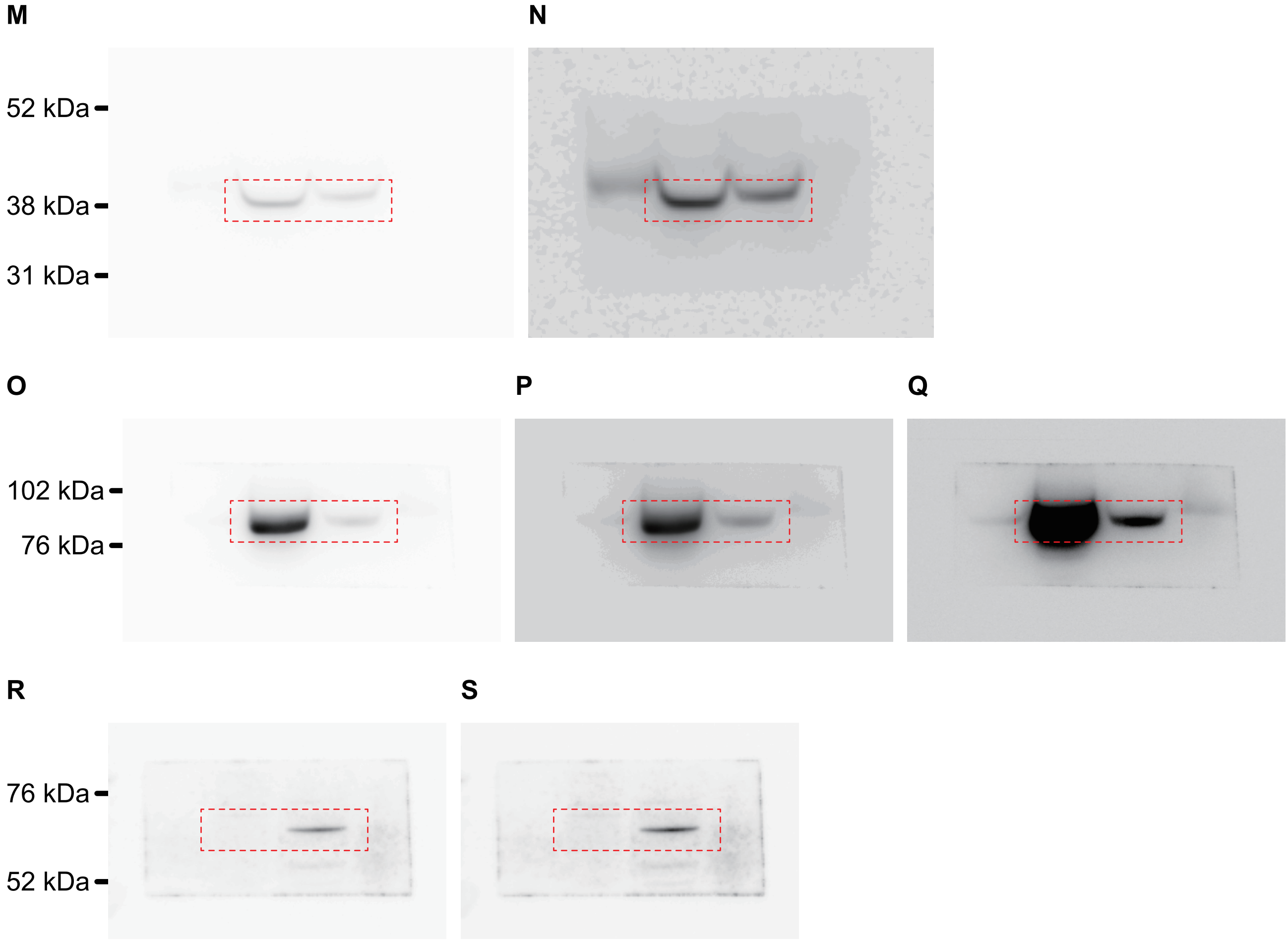

Figure S9 Whole western blots from Figure 1G in the manuscript. Amersham full-range rainbow molecular-weight marker was used to calculate sample molecular weights and sizes in kDa are indicated. Panels A, C, E, G, I, L, M, O, and R show the original images used for the detection of each protein. The brightness and contrast were not changed in the process of acquiring this images. Panels B, D, F, H, J, K, N, P, Q, and S are the same images as A, C, E, G, I, L, M, O, and R, but with different brightness, contrast, and exposure. Red dotted lines show the cropping locations.

Figure S10

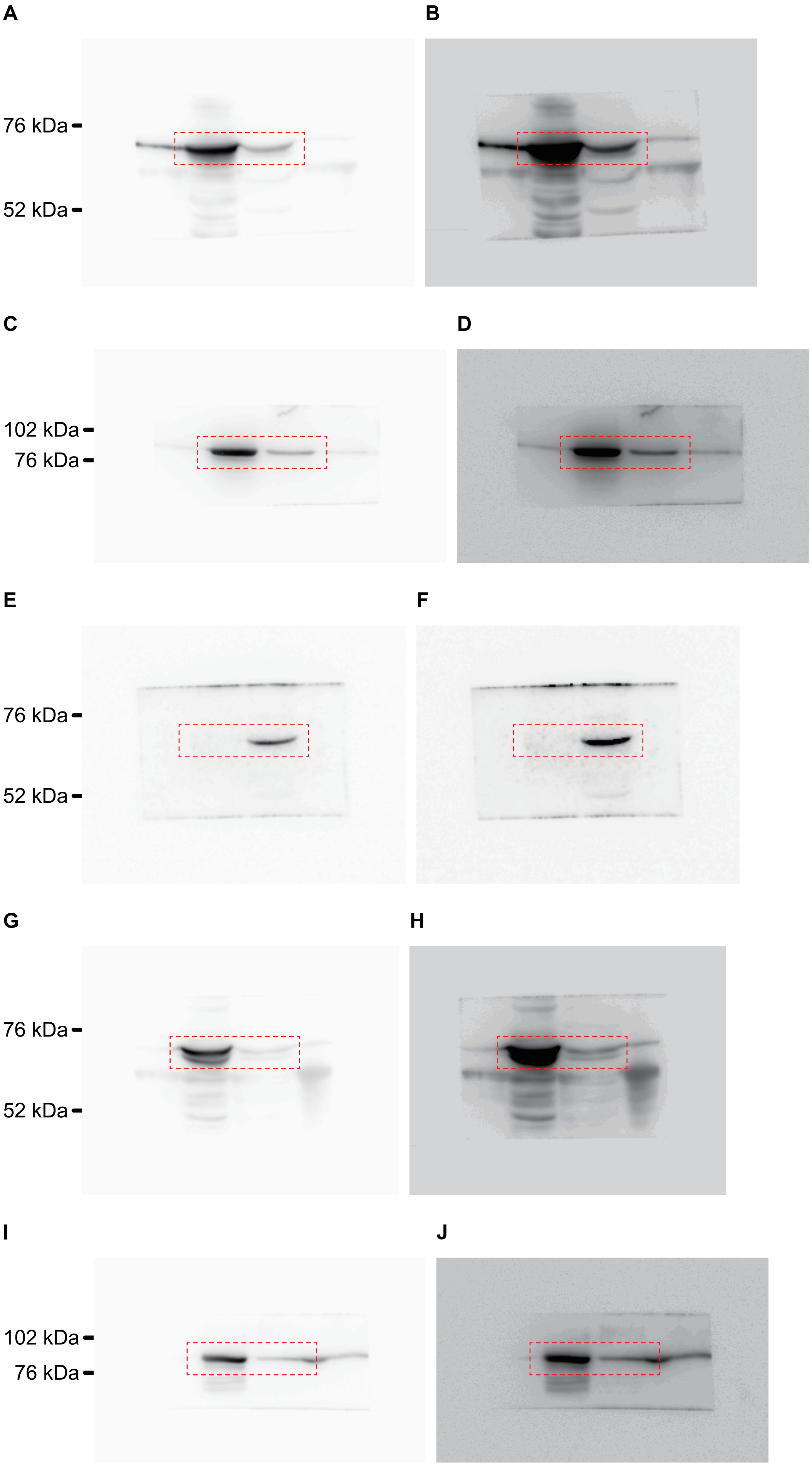
A
B
76 kDa
52 kDa
C
D
102 kDa
76 kDa
E
F
76 kDa
52 kDa
G
H
76 kDa
52 kDa
I
J
102 kDa
76 kDa

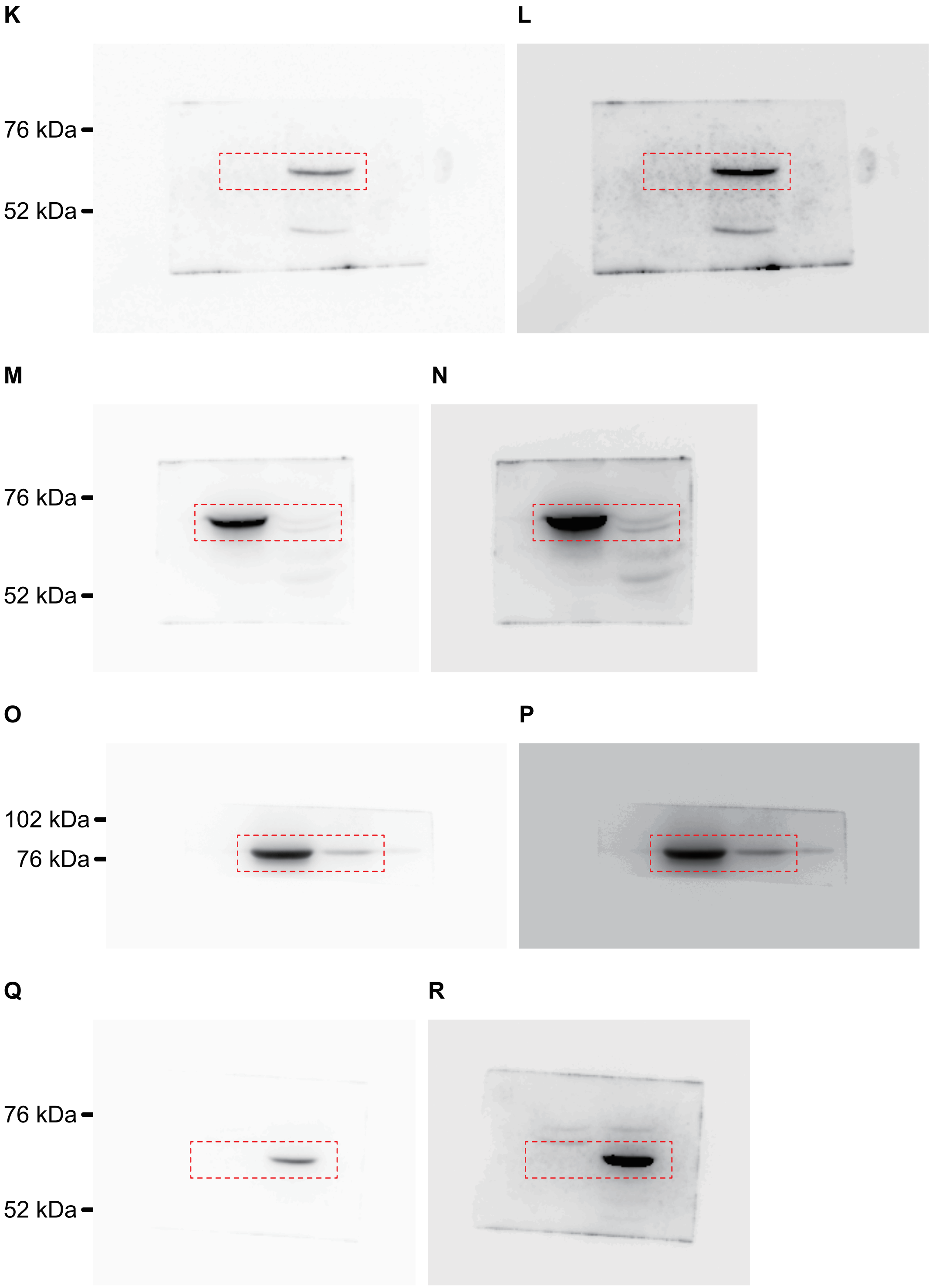

Figure S10 Whole western blots from Figure 2C in the manuscript. Amersham full-range rainbow molecularweight marker was used to calculate sample molecular weights and sizes in kDa are indicated. Panels A, C, E, G, I, K, M, O, and Q show the original images used for the detection of each protein. The brightness and contrast were not changed in the process of acquiring this images. Panels B, D, F, H, J, L, N, P, and R are the same images as A, C, E, G, I, K, M, O, and Q, but with different brightness, contrast, and exposure. Red dotted lines show the cropping locations.

Figure S11

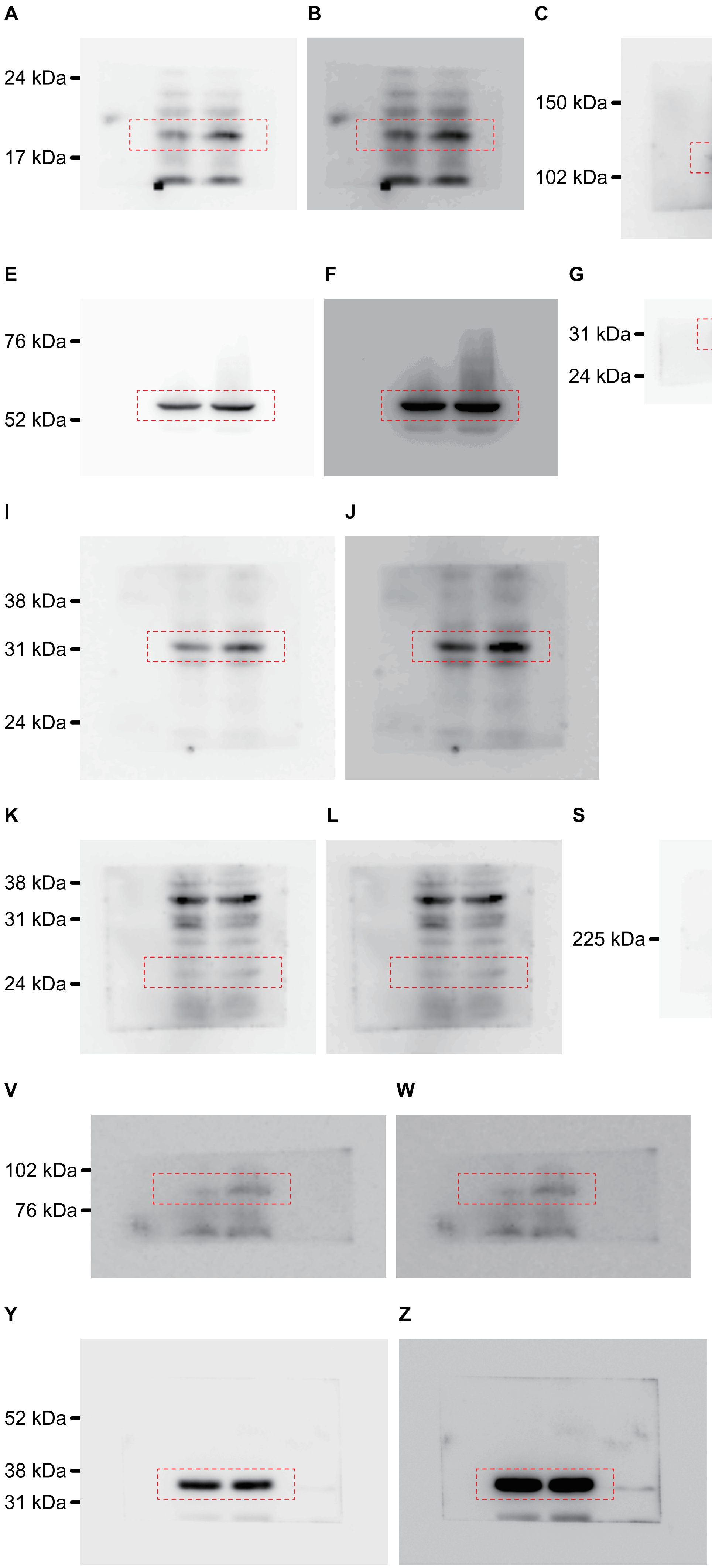

Figure S11 Whole western blots from Figure 5D in the manuscript. Amersham full-range rainbow molecularweight marker was used to calculate sample molecular weights and sizes in kDa are indicated. Panels A, C, E, G, I, K, S, V, and Y show the original images used for the detection of each protein. The brightness and contrast were not changed in the process of acquiring this images. Panels B, D, F, H, J, L, T, W, and Z are the same images as A, C, E, G, I, K, S, V, and Y, but with different brightness, contrast, and exposure. Red dotted lines show the cropping locations.

Figure S12

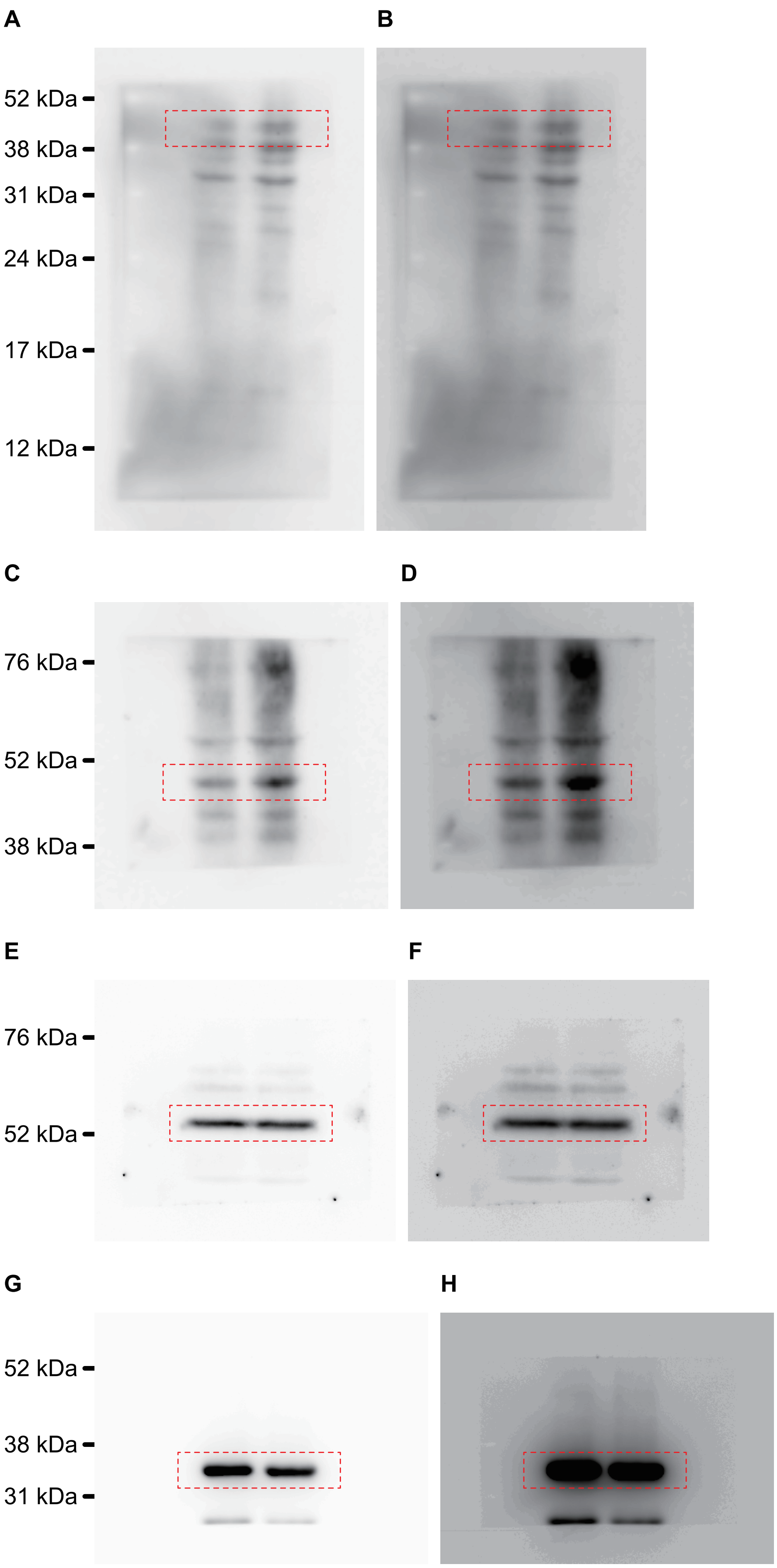

Figure S12 Whole western blots from Figure 5F in the manuscript. Amersham full-range rainbow molecularweight marker was used to calculate sample molecular weights and sizes in kDa are indicated. Panels A, C, E, and G show the original images used for the detection of each protein. The brightness and contrast were not changed in the process of acquiring this images. Panels B, D, F, and H are the same images as A, C, E, and G, but with different brightness, contrast, and exposure. Red dotted lines show the cropping locations.

Figure S13

A

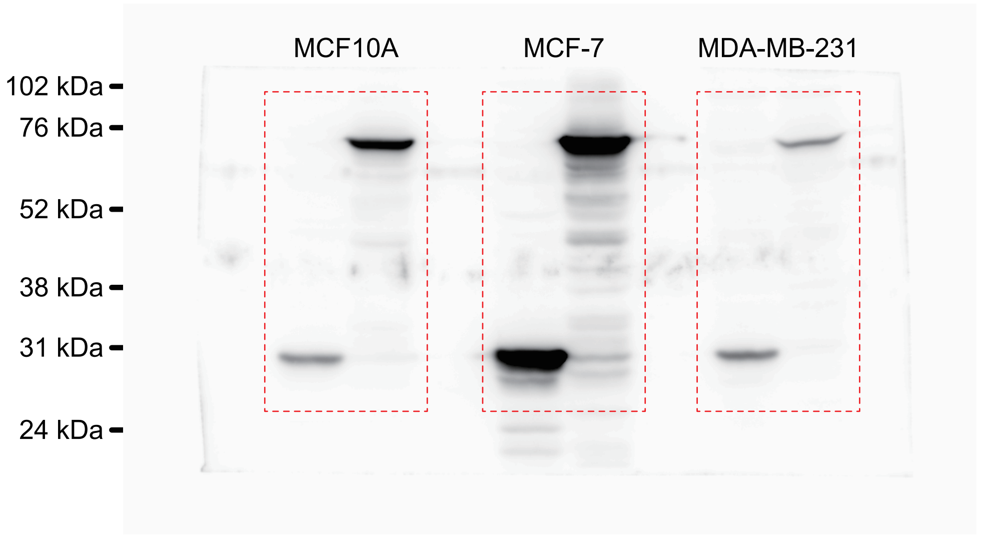

B

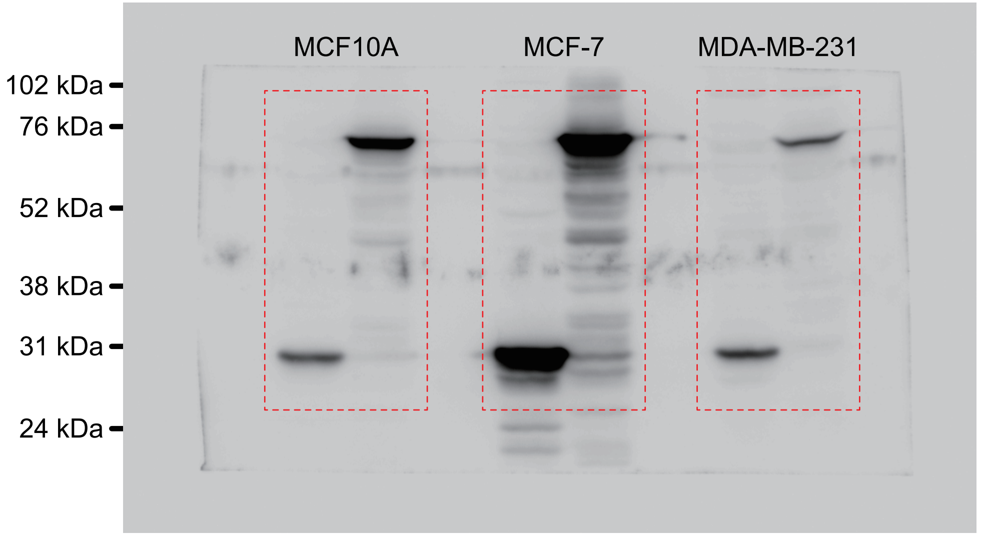

C

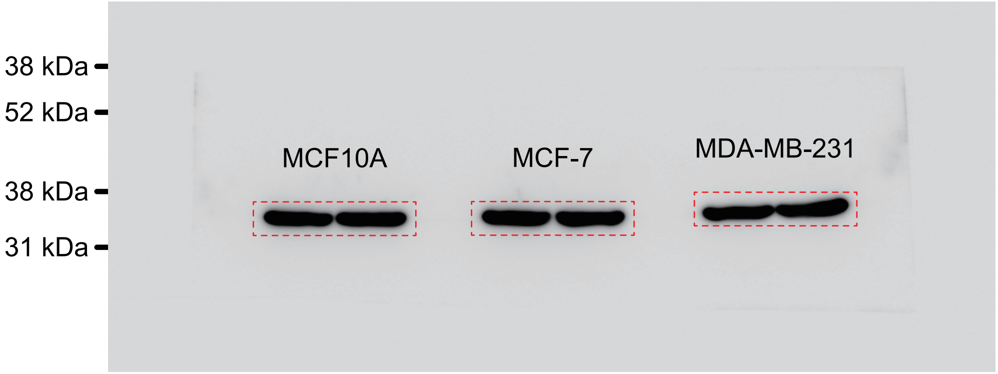

D

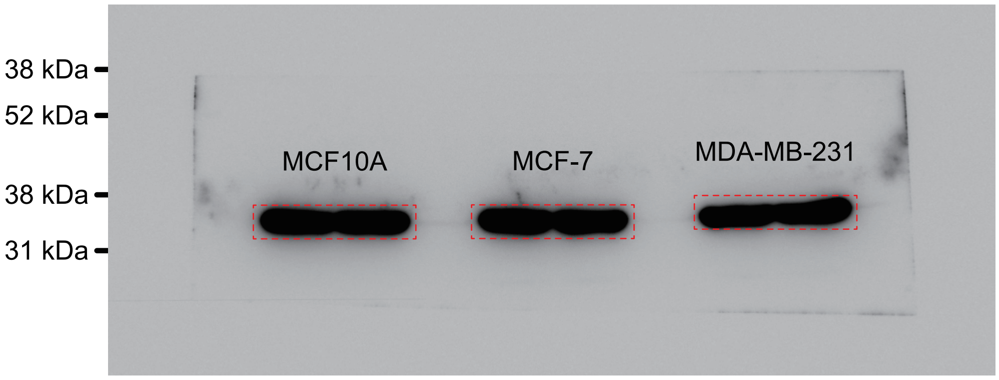

Figure S13 Whole western blots from Supplementary Figure S2D in the manuscript. Amersham full-range rainbow molecular-weight marker was used to calculate sample molecular weights and sizes in kDa are indicated. Panels A, and C show the original images used for the detection of each protein. The brightness and contrast were not changed in the process of acquiring this images. Panels B, and D are the same images as A, and C, but with different brightness, contrast, and exposure. Red dotted lines show the cropping locations.

Figure S14

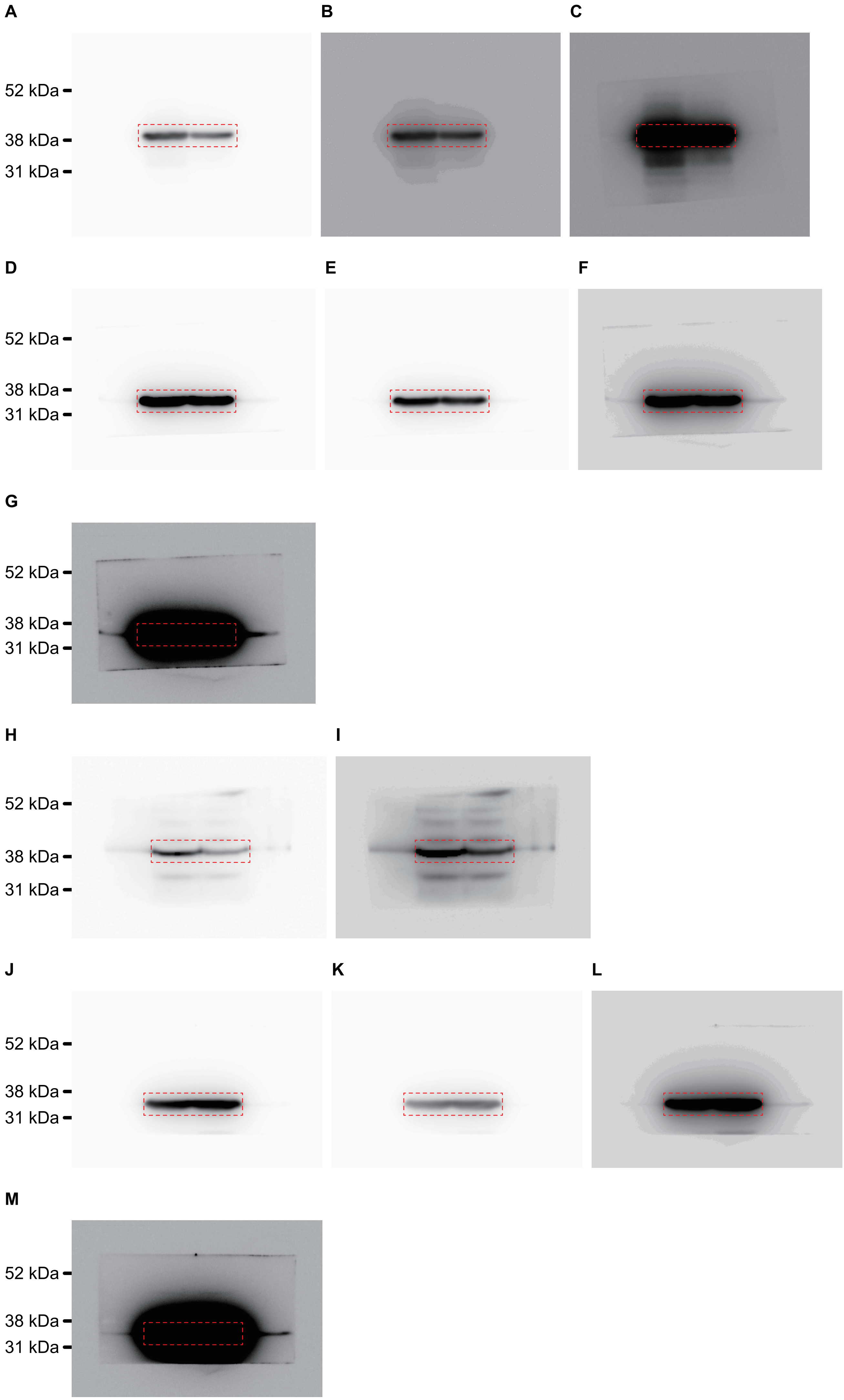
A
B
C
52 kDa
38 kDa
31 kDa
D
E
F
52 kDa
38 kDa
31 kDa
G
52 kDa
38 kDa
31 kDa
H
I
52 kDa
38 kDa
31 kDa
J
K
L
52 kDa
38 kDa
31 kDa
M
52 kDa
38 kDa
31 kDa

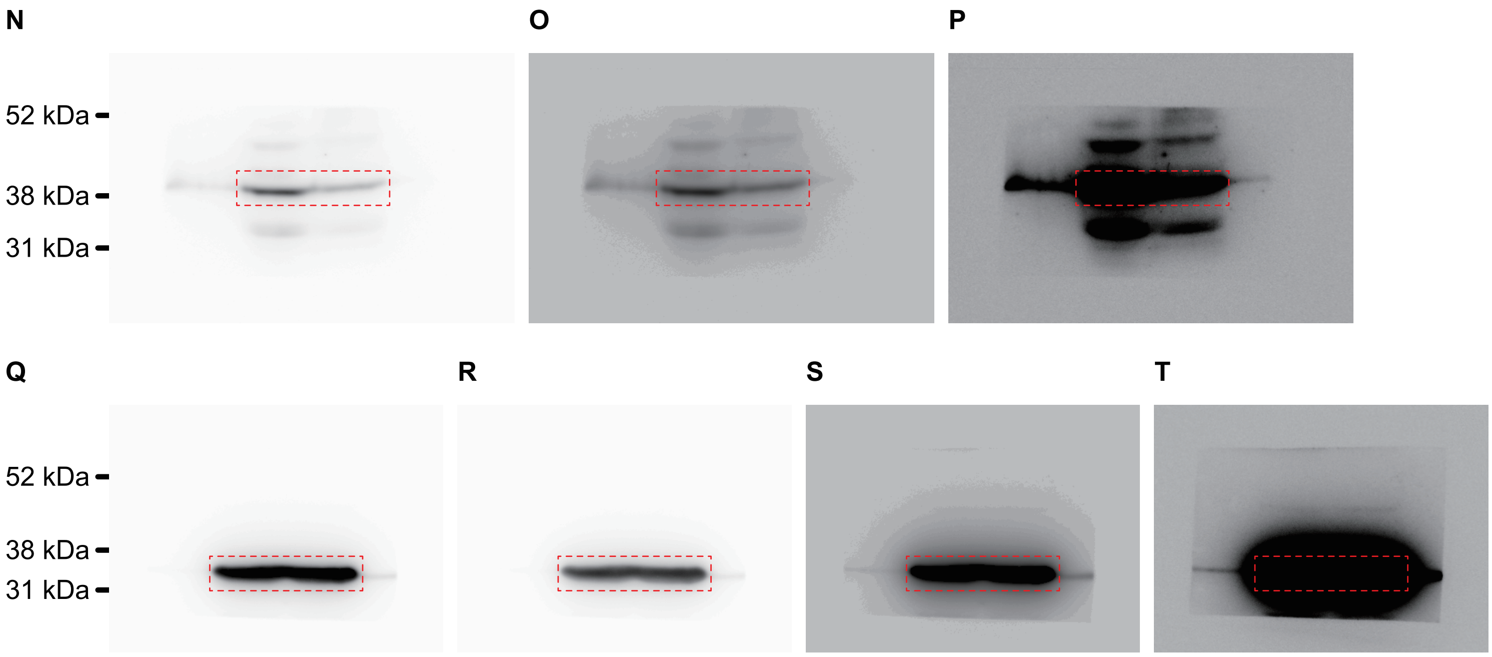

Figure S14 Whole western blots from Supplementary Figure S3D in the manuscript. Amersham full-range rainbow molecular-weight marker was used to calculate sample molecular weights and sizes in kDa are indicated. Panels A, D, H, J, N, and Q show the original images used for the detection of each protein. The brightness and contrast were not changed in the process of acquiring this images. Panels B, C, E, F, G, I, K, L, M, O, P, R, S, and T are the same images as A, D, H, J, N, and Q, but with different brightness, contrast, and exposure. Red dotted lines show the cropping locations.

Figure S15

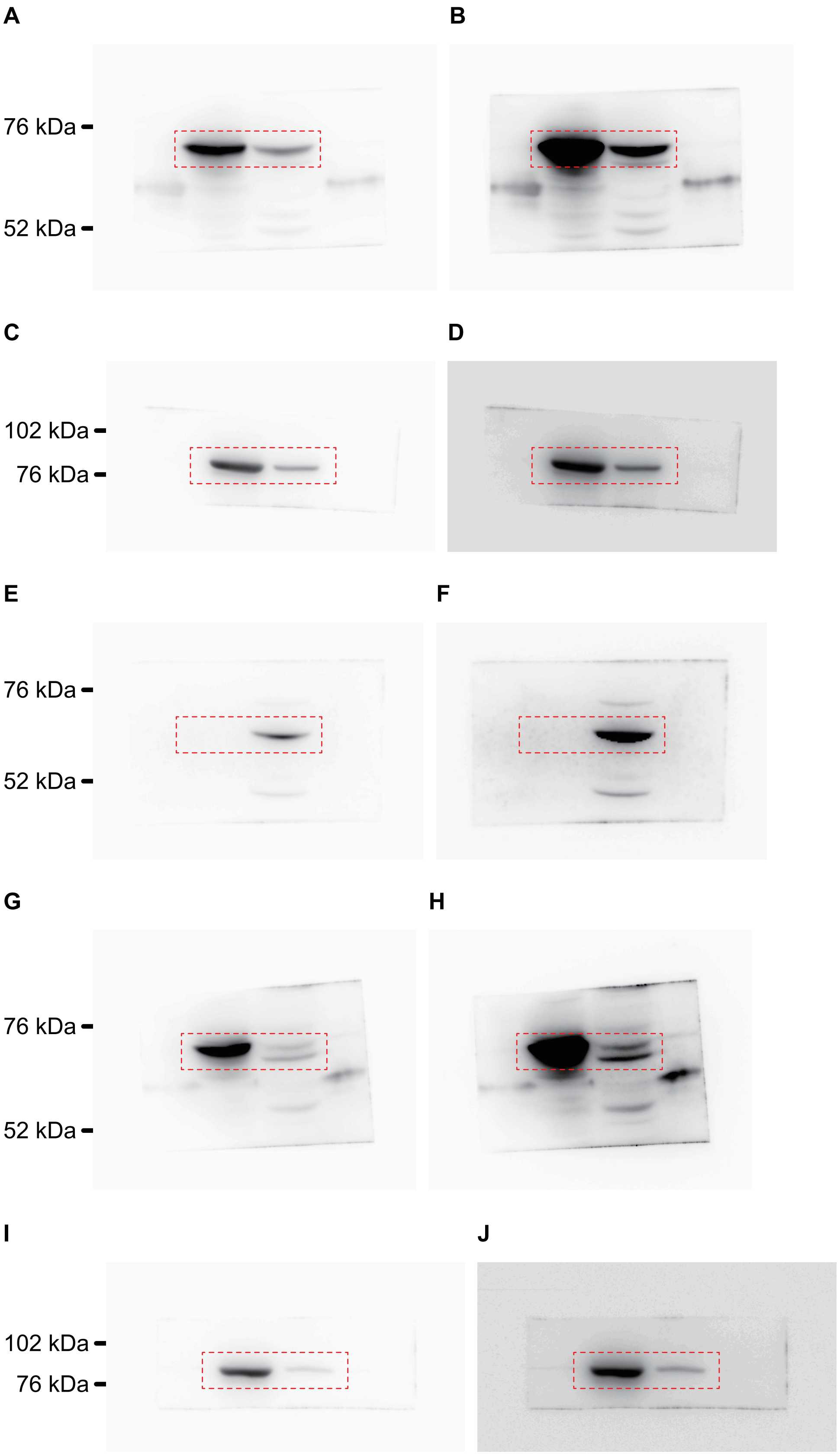

A
B
76 kDa
52 kDa
C
D
102 kDa
76 kDa
E
F
76 kDa
52 kDa
G
H
76 kDa
52 kDa
I
J
102 kDa
76 kDa

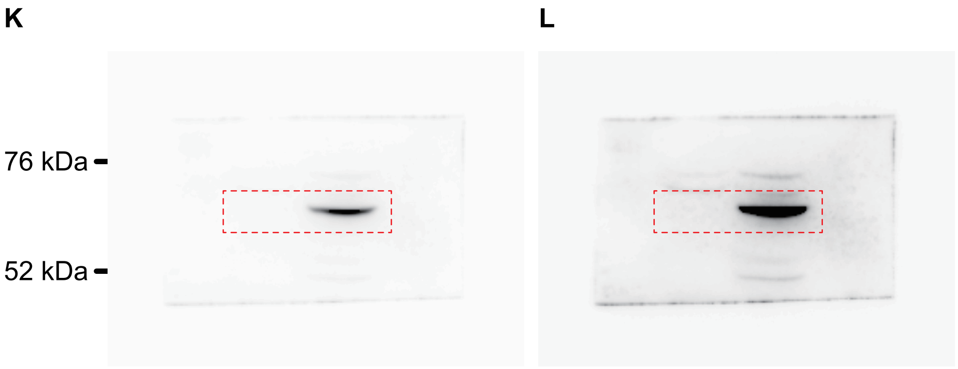

Figure S15 Whole western blots from Supplementary Figure 4C in the manuscript. Amersham full-range rainbow molecularweight marker was used to calculate sample molecular weights and sizes in kDa are indicated. Panels A, C, E, G, I, and K show the original images used for the detection of each protein. The brightness and contrast were not changed in the process of acquiring this images. Panels B, D, F, H, J, and L are the same images as A, C, E, G, I, and K, but with different brightness, contrast, and exposure. Red dotted lines show the cropping locations.

Figure S16

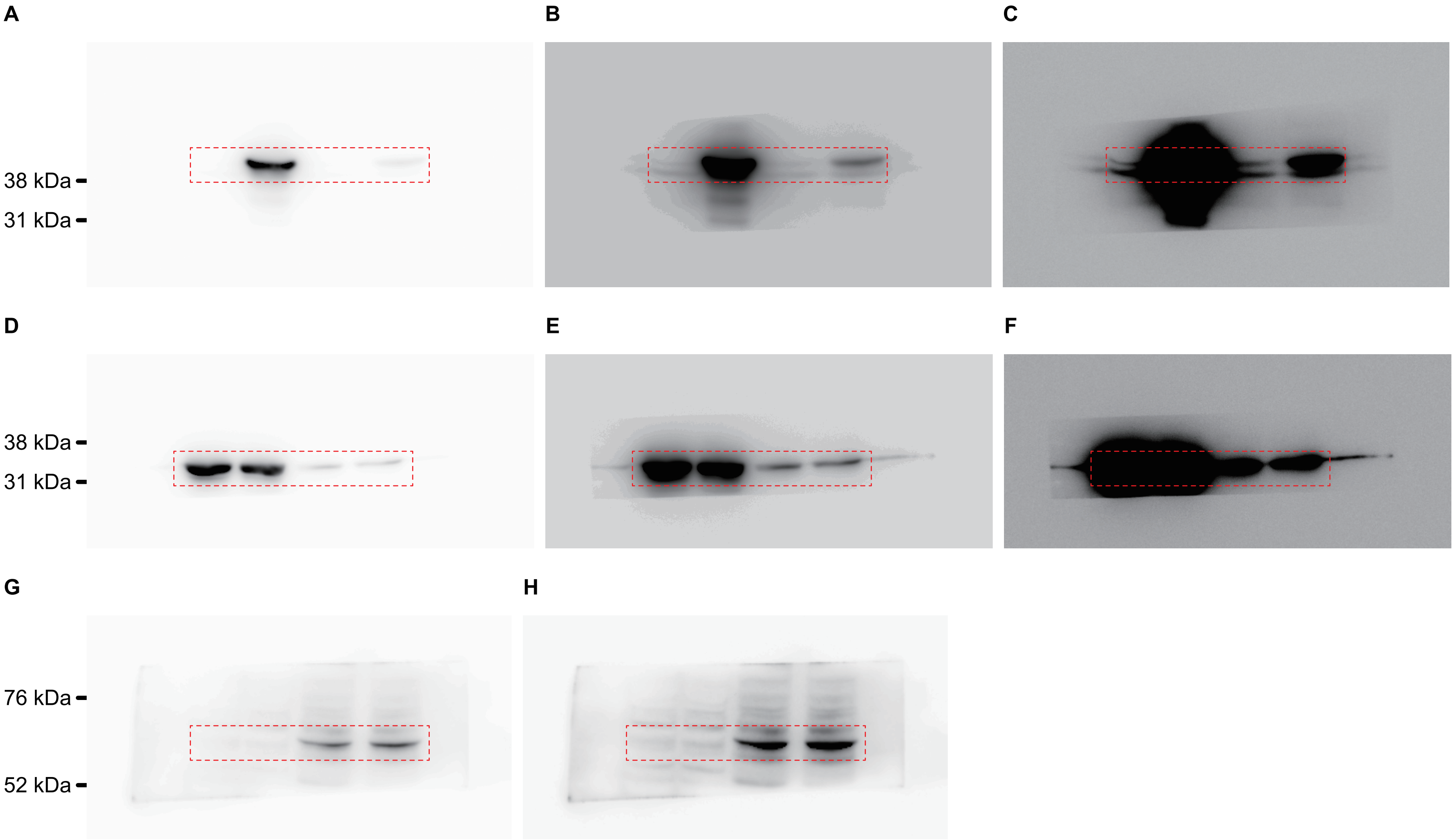

Figure S16 Whole western blots from Supplementary Figure S5B and S5C in the manuscript. Amersham full-range rainbow molecularweight marker was used to calculate sample molecular weights and sizes in kDa are indicated. Panels A, D, and G show the original images used for the detection of each protein. The brightness and contrast were not changed in the process of acquiring this images. Panels B, C, E, F, and H are the same images as A, D, and G, but with different brightness, contrast, and exposure. Red dotted lines show the cropping locations.
